# Menstrual cycle length identifies perinatal risk information beyond routine metabolic antenatal screening: a retrospective cohort study of 17,792 deliveries

**DOI:** 10.3389/fendo.2026.1888560

**Published:** 2026-08-03

**Authors:** Qian Xia, Hong Mei, Dongmei Cao, Jingjing Rao, Feng Tang, Yun Xiang, Changzhen Li

**Affiliations:** 1 Department of Laboratory Medicine, Wuhan Children’s Hospital (Wuhan Maternal and Child Healthcare Hospital), Tongji Medical College, Huazhong University of Science & Technology, Wuhan, China; 2 Institute of Maternal and Child Health, Wuhan Children’s Hospital (Wuhan Maternal and Child Healthcare Hospital), Tongji Medical College, Huazhong University of Science & Technology, Wuhan, China

**Keywords:** Additive interaction, gestational diabetes, low birth weight, menstrual cycle length, perinatal risk stratification, population attributable fraction, preterm birth

## Abstract

**Background:**

Preterm birth (PTB) and low birth weight (LBW) are leading causes of neonatal mortality and long-term morbidity. In China, the high prevalence of gestational diabetes mellitus (GDM) and thyroid dysfunction (TF) creates a metabolically complex obstetric environment in which identifying risk markers that add value beyond existing screening is a priority. Menstrual cycle length is routinely available, yet whether it provides risk information non-redundant with GDM and TF has not been rigorously examined.

**Methods:**

Retrospective cohort of 17,792 consecutive singleton deliveries at a tertiary centre in Wuhan, China (2020–2024). Cycle length was recorded at intrapartum admission: short (<25d), normal (25–35d; reference), or long (>35d). Primary outcomes were PTB (<37 weeks) and LBW (<2,500 g). Three nested logistic models were fitted; stability across models was used to assess whether GDM and TF acted as strong confounders. Restricted cubic splines, additive interaction (RERI), population-attributable fractions (PAF), and five sensitivity analyses were used, with Benjamini–Hochberg FDR correction.

**Results:**

Long cycle (>35d; n = 1,810, 10.2%) was independently associated with PTB (aOR 1.36, 95% CI 1.12–1.64; FDR *p* = 0.006) and LBW (aOR 1.46, 1.18–1.78; FDR *p* = 0.002) after full adjustment for GDM and TF. Estimates were stable across progressive adjustment (PTB 1.34→1.36; LBW 1.42→1.46), indicating GDM and TF were not strong confounders. RERI for long cycle × GDM/TF crossed zero for both outcomes, excluding meaningful synergism. RCS showed a monotonic increase from ~30 days. PAF: PTB 3.1% (0.9–5.4%); LBW 3.9% (1.2–6.4%). The birth weight reduction (*β* = −33 g) attenuated at term (*β* = −8 g, NS), but long cycle was associated with higher odds of small-for-gestational-age (aOR 1.39, 1.11–1.73), including at term, indicating both earlier delivery and impaired fetal growth. Period length showed no association.

**Conclusions:**

Long menstrual cycle length was associated with PTB and LBW, not materially attenuated by adjustment for GDM and TF, consistent with prognostic information non-redundant with metabolic screening; however, stable estimates cannot exclude residual confounding or establish an independent pathway. Because cycle length reflects the pre-pregnancy ovulatory interval, any clinical value would lie in collecting menstrual history before conception or in early pregnancy. These findings are hypothesis-generating and require prospective validation in community-based cohorts.

## Introduction

Preterm birth (PTB, <37 weeks) and low birth weight (LBW, <2,500 g) remain the leading causes of neonatal mortality and long-term neurodevelopmental morbidity worldwide. In 2020, an estimated 13.4 million newborns were born preterm – about one in ten live births – with no measurable global reduction over the previous decade (1). In China, increasing rates of advanced maternal age, epidemic gestational diabetes mellitus (GDM; prevalence 14–21% nationally, with substantially higher rates reported at tertiary obstetric referral centers) (2, 3), and a high burden of thyroid dysfunction (8–35% depending on diagnostic criteria) (4, 5) have created an unusually complex obstetric risk landscape. A central challenge in this context is to identify risk markers that provide predictive value beyond the metabolic comorbidities already systematically screened for during antenatal care.

The American College of Obstetricians and Gynecologists (ACOG) formally endorsed the menstrual cycle as a “fifth vital sign” of systemic reproductive and metabolic health in 2015 (6). The 2023 International Evidence-Based PCOS Guideline, co-endorsed by 39 organizations across 71 countries (7), identifies cycle length as a key indicator of reproductive-metabolic status. Long or irregular cycles (>35 days) are associated with insulin resistance (8, 9), an elevated lifetime risk of type 2 diabetes (10, 11) and gestational diabetes mellitus (GDM) (12), premature mortality (13), hypertension (14), and cardiovascular disease (15–19). These associations demonstrate that menstrual cycle phenotype reflects an independent dimension of physiological health – distinct from, but correlated with, metabolic comorbidities. A 2024 Nature Communications systematic review (73 studies, 92,881 offspring) found that maternal PCOS is independently associated with increased odds of preterm birth (PTB), fetal growth restriction, and low birth weight (LBW) (20). A Swedish nationwide register-based study of over one million births confirmed a more than two-fold increased risk of extremely preterm birth in women with PCOS, predominantly of spontaneous onset (21).

Despite this convergent evidence, a fundamental question remains unanswered: does menstrual cycle length predict adverse perinatal outcomes independently of, and in addition to, GDM and thyroid dysfunction – the two metabolic comorbidities most intensively monitored in Chinese obstetric practice? This distinction is not trivial. If the association between long cycle length and PTB is fully mediated by metabolic comorbidities, including cycle length in antenatal risk assessment would provide no value beyond what GDM and thyroid screening already provide. Conversely, if cycle length predicts PTB and LBW beyond measured metabolic comorbidities – and without synergistic amplification – it offers risk information not captured by metabolic screening, representing a potentially actionable perinatal signal that complements current screening paradigms. We do not assume this constitutes a causally distinct “non-metabolic” axis, as establishing that would require mechanistic data not available in the present study. We framed these questions as a problem of incremental, non-redundant prognostic information rather than causal mechanism, while making the assumed causal positions of the key covariates — pre-pregnancy and admission body-mass index, GDM, thyroid dysfunction, and polycystic ovary syndrome — explicit in our analytical framework. The potential biological rationale linking long cycles to preterm delivery involves luteal-phase insufficiency and relative progesterone deficiency, consistent with interventional evidence that vaginal progesterone supplementation reduces PTB risk in women with a short cervix (PREGNANT trial; RR 0.55, 95% CI 0.33–0.92) (22); however, our observational study cannot directly test this mechanism.

The only large prospective study directly examining cycle length and perinatal outcomes – the Project Viva cohort (Soria-Contreras et al., 2022; n = 2,046)—reported a relative risk of approximately 2.04 for preterm birth in women with long or irregular cycles (23). However, that cohort had a substantially lower metabolic comorbidity burden than Chinese tertiary obstetric populations, and gestational diabetes mellitus and thyroid dysfunction were not simultaneously adjusted for. Three *a priori* research questions – documented in the analysis plan before data extraction – guided our analysis: (i) Does the association between long cycle length and preterm birth or low birth weight persist after comprehensive simultaneous adjustment for both gestational diabetes mellitus and thyroid dysfunction in a high-burden Chinese cohort? (ii) Do menstrual cycle length and metabolic comorbidities provide non-redundant risk information without superadditive interaction (relative excess risk due to interaction ≈ 0)? (iii) Is the association specific to cycle length (ovulatory interval) rather than period length (endometrial shedding duration), which would serve as a supportive but underpowered dimension-specificity comparison? We addressed these questions in a retrospective cohort of 17,792 consecutive singleton deliveries, with period length as a pre-defined internal comparison dimension. As this cohort was drawn from a single tertiary referral centre with an unusually high metabolic-comorbidity burden, the resulting estimates should be interpreted as characterising a high-risk, metabolically enriched obstetric population rather than a general community sample, a point examined further among the study’s limitations.

## Methods

### Study design and population

We conducted a retrospective cohort study using electronic health records (EHR) from Wuhan Children’s Hospital (Wuhan Maternal and Child Healthcare Hospital), a tertiary obstetric referral center in Wuhan, Hubei, China, from January 2020 to December 2024. Of 18,709 consecutive deliveries documented in the EHR during the study period, 17,792 met all eligibility criteria after pre-specified sequential exclusions (total excluded: n = 917; 95.1% of the original cohort; **Supplementary Figure 1**). Exclusions were applied in the following order: (1) multiple gestation (n = 332), as multiple pregnancy directly distorts birth weight and gestational age distributions; (2) PCOS (n = 245), ascertained via a four-source approach – ICD-10 coded diagnosis (E28.2) in any inpatient or outpatient encounter in the hospital information system, clinician diagnosis documented during antenatal care, patient self-report at the booking visit capturing external diagnoses, and structured clinical note review – because oligomenorrhea is a cardinal PCOS diagnostic feature rather than a metabolic function indicator, and its retention would introduce severe confounding of the primary exposure; routine first- and second-trimester ultrasound was performed for all women, and clinically evident polycystic ovarian morphology would have prompted diagnostic coding captured by the above criteria; (3) pre-existing chronic hypertension (n = 287), indistinguishable from gestational hypertension; (4) pituitary disease, including prolactinoma and hyperprolactinemia (n = 8), constituting an independent HPO-axis cause of menstrual phenotype; (5) fetal chromosomal anomaly (n = 29); (6) systemic lupus erythematosus and antiphospholipid syndrome (n = 10); and (7) maternal congenital heart disease (n = 6). The final analytic cohort comprised 17,792 women.

### Exposure ascertainment: three a priori-defined menstrual dimensions

All menstrual phenotype data were collected by trained midwives using a standardized structured interview protocol at the intrapartum admission visit (i.e., upon presentation to the labor ward for delivery), before formal outcome recording was completed. Interviewers asked two pre-specified questions: “What is your usual menstrual cycle length – that is, the number of days from the first day of one period to the first day of the next?” and “How many days does your period typically last?” Responses were entered directly as integer values into discrete numeric fields within the hospital’s electronic health record system (HIS); the system flagged entries outside plausible ranges (cycle length 15–90 days; period length 1–15 days) for interviewer verification before submission. This structured-field approach eliminates free-text transcription errors and ensures uniform data capture across the five-year study period. The question referred to the woman’s usual pre-pregnancy cycle, and the same standardised protocol was administered to every admission irrespective of gestational age, so exposure ascertainment did not differ between preterm and term deliveries. The structured field did not capture recent hormonal contraception, ovulation induction, or luteal-phase support, which therefore could not be separately identified or adjusted for; pregnancies conceived by assisted reproductive technology were examined in a dedicated sensitivity analysis (SA4, n = 16,909), in which the long-cycle associations were unchanged. Three dimensions were defined prior to data extraction:

Dimension 1 – Cycle length: usual cycle duration (day 1 to next day 1), categorized as short (<25 days), normal (25–35 days; reference), or long (>35 days). This dimension represents the ovulatory interval and is the primary exposure of interest.

Dimension 2 – Menarche age: categorized as ≤11 years, 12–13 years (reference), 14–15 years, or ≥16 years.

Dimension 3 – Period length (endometrial dimension; *a priori* comparison): duration of menstrual bleeding per cycle, categorized as short (<3 days), normal (3–7 days; reference), or long (>7 days). This dimension was defined *a priori* as a dimension-specificity comparison: if cycle length (ovulatory interval), but not period length (endometrial shedding), predicts PTB and LBW, this constitutes a supportive but underpowered dimension-specificity comparison, consistent with the interpretation that the association is specific to the timing dimension of the menstrual phenotype rather than to menstrual health in general. We caution that the period-length analysis was substantially underpowered (**Supplementary Table 1**); this comparison is hypothesis-generating rather than confirmatory.

### Outcome definitions

Gestational age was confirmed by first-trimester ultrasound crown-rump length (CRL) at 11–14 weeks according to institutional standards; LMP-based dating was not used as the primary method. Primary outcomes were: (1) PTB – delivery before 37 completed weeks; (2) LBW – birthweight less than 2,500 g. PTB cases were further classified as spontaneous or indicated on the basis of ICD-10-coded delivery and obstetric-complication diagnoses (spontaneous: preterm premature rupture of membranes or spontaneous labour onset in the absence of documented iatrogenic indicators; indicated: delivery for severe pre-eclampsia, HELLP, placental abruption, or fetal growth restriction); cases that could not be distinguished from the EHR text were coded as uncertain. This classification was *post-hoc* and the results are reported as exploratory (**Supplementary Table 2**). Secondary outcomes included gestational hypertension (new-onset BP ≥140/90 mmHg after 20 weeks), macrosomia (birthweight greater than 4,000 g), amniotic fluid abnormality, and fetal distress. To distinguish impaired fetal growth from earlier delivery, we additionally derived birthweight-for-gestational-age z-scores using the INTERGROWTH-21st Newborn Birth Weight Standards (24). Gestational age for this analysis was derived from the last menstrual period and date of delivery. Small-for-gestational-age (SGA) was defined as a birthweight z-score below the 10th centile, severe SGA/fetal growth restriction (FGR) as below the 3rd centile, and large-for-gestational-age (LGA) as above the 90th centile. The INTERGROWTH-21st newborn standard spans 33 + 0 to 42 + 6 weeks; infants born before 33 weeks (n = 219) fell outside the standard and were set to missing for the size-for-gestational-age analysis. All outcomes were ascertained from ICD-10-coded EHR records and verified by attending obstetricians.

### Covariates and comorbidity definitions

The fully adjusted model (M3) included maternal age (years, continuous), parity (primipara/para 1/para ≥2), gravidity (continuous), admission BMI (kg/m^2^, continuous), year of admission (2020–2024, categorical), GDM status, and thyroid dysfunction (TF) status. Pre-pregnancy BMI is the theoretically preferred confounder because it precedes both exposure ascertainment and pregnancy outcomes. However, due to 58.3% missing data, the primary analysis used admission BMI as a pragmatic proxy (available for 98.4% of women). Logistic regression of predictors of pre-pregnancy BMI missingness confirmed that thyroid dysfunction (OR 18.48) and parity were the strongest predictors of missing pre-pregnancy BMI, while long menstrual cycle was not associated with missingness (OR 1.04, *p* = 0.578), arguing against exposure-related selection bias (**Supplementary Table 3**). We considered BMI as a potential confounder–mediator hybrid: admission BMI was adjusted for to minimize confounding by maternal adiposity, while sensitivity analyses using pre-pregnancy BMI were conducted to assess robustness to alternative temporal specifications. We did not interpret BMI-adjusted estimates as total causal effects; all M3 aOR estimates should therefore be interpreted as conditional associations rather than total effects. The causal position of admission BMI relative to the other analysis variables is illustrated in **Supplementary Figure 2**. Adjustment for admission BMI may produce conservative (downward-biased) effect estimates where it acts as a mediator. The MICE analysis using imputed pre-pregnancy BMI served as the primary sensitivity analysis for BMI measurement strategy (**Supplementary Table 4**). To make the dependence of the findings on BMI handling explicit, we additionally compared four models for the primary outcomes that were identical except for the BMI term: (i) no BMI adjustment; (ii) admission BMI (the primary specification); (iii) pre-pregnancy BMI in complete cases; and (iv) pre-pregnancy BMI imputed by MICE (**Supplementary Table 5**). GDM was diagnosed by the one-step 75 g OGTT per IADPSG criteria (25). Thyroid dysfunction was diagnosed by attending endocrinologists following the 2019 Chinese Thyroid Society guidelines and 2017 ATA recommendations, which specify pregnancy-specific TSH reference ranges (first trimester upper limit 4.0 mIU/L; second/third trimester 4.0 mIU/L) with FT4 used to distinguish overt from subclinical disease. Subtype classification was derived from ICD-10-coded hospital diagnoses and clinical documentation: clinical hypothyroidism n = 4,856 (83.7%), hyperthyroidism n = 378 (6.5%), thyroiditis n = 311 (5.4%), other/unspecified n = 258 (4.4%). The clinical hypothyroidism category (ICD code: O99.2/hypothyroidism complicating pregnancy) includes all cases receiving levothyroxine treatment during pregnancy–encompassing both overt hypothyroidism (elevated TSH with low FT4) and subclinical hypothyroidism (elevated TSH with normal FT4 meeting treatment thresholds per gestational age-specific criteria); review of clinical notes identified explicit subclinical terminology in approximately 11% of these cases, with the remainder meeting TSH-based treatment criteria without overt FT4 suppression. This classification reflects Chinese tertiary obstetric practice, where systematic first-trimester thyroid screening and treatment of subclinical hypothyroidism is routine. Sensitivity analysis SA5, restricted to the clinical hypothyroidism subgroup (n = 16,562), confirmed directionally consistent findings (**Supplementary Table 6**), supporting the robustness of primary results to TF subtype composition. The simultaneous inclusion of both GDM and TF in M3 was the critical test of cycle-length independence: if either comorbidity explained the cycle-length associations, the aOR would be substantially attenuated from M2 to M3.

### Statistical analysis

Of the 17,792 eligible pregnancies, cycle length data were available for 17,624 (99.1%); the 168 women (0.9%) with missing cycle length were excluded from cycle length analyses on a complete-case basis. Given the low proportion of missing data, substantial selection bias is considered unlikely. Of the 17,772 women with period length data available, 339 (1.9%) had long periods (>7 days); of these, 335 had complete covariate data and were included in period length complete-case regression analyses. The overall pattern of variable missingness is shown in **Supplementary Figure 3**; pre-pregnancy BMI had the highest missingness (58.3%), motivating the use of admission BMI as the primary BMI proxy and MICE imputation as a sensitivity analysis. Three nested logistic regression models were used per exposure–outcome pair: M1 (crude), M2 (adjusted for age, parity, admission year), and M3 (fully adjusted, including GDM and TF). The M3 complete-case analysis included 17,336 women (17,624 with cycle length data minus 288 missing admission BMI or other covariates). The primary inferential question was whether M3 aORs differed materially from M1 or M2; stability of the association across models was interpreted as evidence that GDM and TF did not act as strong confounders, rather than as evidence of an independent causal pathway. Multiple comparisons within each exposure dimension (8 tests: 2 exposure categories × 4 primary outcomes) were controlled using Benjamini–Hochberg (BH) FDR correction (26). A combined FDR across all 24 tests confirmed robustness (**Supplementary Table 7**): long cycle → PTB (combined FDR p = 0.015) and → LBW (combined FDR p = 0.006) remained significant. Dose–response analyses used restricted cubic splines (RCS; 5 knots) via the rms package (27), with reference values: cycle 28 days, menarche 13 years, period 5 days. For cycle length, knots were placed at the rms default 5th, 27.5th, 50th, 72.5th, and 95th percentiles (26, 29, 30, 30, and 40 days; the inner knots coincide because the distribution is sharply peaked, with an interquartile range of 29–30 days). The tails contained 553 women (≤26 days, lower tail) and 1,065 women (≥40 days, upper tail), with 637 women (3.6%) at ≥45 days – the range relevant to the residual-PCOS assessment. Cycle-length values were retained as recorded within the pre-specified plausible range (15–90 days; observed maximum 60 days) and were not winsorised, so the spline tails reflect the actual data rather than truncated values. Additive interaction was quantified by RERI and AP using the delta method (interactionR package) following VanderWeele and Knol (28). RERI ≈ 0 was defined *a priori* as a test of non-synergistic independence: absence of superadditive interaction between cycle length and metabolic comorbidities would be consistent with these constituting independent risk dimensions rather than components of the same amplifying causal pathway. To assess the informativeness of RERI findings, *post hoc* power was estimated from delta-method standard errors for each outcome–modifier pair: for PTB × GDM (SE = 0.29), power to detect RERI = 0.30 was approximately 17.7% and to detect RERI = 0.50 was approximately 40.3%; full estimates for all eight combinations are reported in **Supplementary Table 8**. Multiplicative interaction was evaluated by likelihood ratio test (LRT). PAF was estimated by counterfactual standardization (999 bootstrap resamples, seed = 42). E-values were computed from M3 estimates following VanderWeele and Ding (29).

Five sensitivity analyses: SA1, GEE clustering on woman ID (results shown in **Supplementary Figure 4**); SA2, pre-pregnancy BMI subset (n = 7,426); SA3, thyroid-dysfunction-free subset (n = 11,753); SA4, non-ART conceptions (n = 16,909; M3 complete-case non-ART subset); SA5, clinical hypothyroidism only (n = 16,562). All analyses used R v4.3.1. Incremental discriminative value was assessed by comparing a base logistic model (M3 covariates) with a full model that additionally included cycle category. AUC differences were evaluated using the DeLong paired test (pROC package). Integrated Discrimination Improvement (IDI) and continuous Net Reclassification Improvement (NRI) were computed following Pencina et al. (30) with 1,000-resample bootstrap 95% confidence intervals (seed = 42). Pre-pregnancy BMI was imputed by chained equations (MICE; m = 20 imputed datasets, predictive mean matching, pooled by Rubin’s rules; **Supplementary Table 4**). The causal assumptions underlying the choice and positioning of covariates — in particular the dual role of admission BMI as a potential mediator or collider rather than a pure confounder — are summarised in a directed acyclic graph (**Supplementary Figure 2**). Model calibration was assessed by grouping women into deciles of predicted probability and plotting observed against mean predicted event rates, with a LOESS smoother and the 45° line of perfect calibration, for the base model (M3 covariates) and the full model (M3 + cycle category) (**Supplementary Figure 5**). Clinical usefulness was evaluated by decision-curve analysis, plotting net benefit across threshold probabilities of 0–30% for both models using the dcurves package (**Supplementary Figure 6**). Calibration and decision-curve analyses were pre-specified as exploratory.

## Results

### Cohort characteristics

Of the 17,792 women included, 6,588 (37.0%) had GDM and 5,803 (32.6%) had thyroid dysfunction. The mean maternal age was 31.3 ± 3.8 years; 11,506 (64.7%) were primiparous; 600 (3.4%) conceived via ART. Baseline characteristics by menstrual cycle length category are shown in **Table 1**. Cycle length data were available for 17,624 (99.1%) women; 168 (0.9%) had missing cycle length data and were excluded from all cycle length analyses. Given this low proportion of missing data, substantial selection bias is unlikely. Among those with data, 1,810 (10.2%) had long cycles (>35 days) and 201 (1.1%) had short cycles (<25 days). The short-cycle group (n = 201) is substantially underpowered (*post hoc* power <35% to detect OR = 1.36 at a PTB baseline rate of 6.1%); all short-cycle results are exploratory and are not included in the primary conclusions. Women with long cycles had higher rates of preterm birth (7.6% vs 5.9% in the normal-cycle group), low birth weight (6.6% vs 4.7%), and gestational hypertension (9.0% vs 7.3%), consistent with the primary findings reported below. GDM prevalence was also higher among women with long cycles (41.4%) than among those with normal cycles (36.6%; **Table 1**), consistent with long cycle length partly reflecting pre-existing insulin resistance shared with the GDM pathway. Period length data were available for 17,772 (99.7%); 339 (1.9%) had long periods (>7 days). Outcome prevalences in the full cohort were: gestational hypertension 7.5%, PTB 6.1%, LBW 4.9%, and macrosomia 3.9%.

**Table 1:** Baseline characteristics stratified by menstrual cycle length category (N = 17,792).

Variable	Overall	Normal (25–35d)	Short (<25d)	Long (>35d)	*P*
N	17,792	15,613	201	1,810	
Maternal age (years)	31.3 ± 3.8	31.3 ± 3.8	32.4 ± 4.3	30.9 ± 3.6	<0.001
Parity, n (%)					0.083
Primipara	11,506 (64.7%)	10,074 (64.5%)	116 (57.7%)	1,210 (66.9%)	
Para 1	5,796 (32.6%)	5,109 (32.7%)	78 (38.8%)	552 (30.5%)	
Para ≥2	490 (2.8%)	430 (2.8%)	7 (3.5%)	48 (2.7%)	
Gravidity	2.0 ± 1.2	2.0 ± 1.2	2.2 ± 1.3	1.9 ± 1.2	0.001
Menarche age (years)	12.9 ± 1.0	12.9 ± 1.0	12.9 ± 0.9	13.0 ± 1.1	<0.001
Cycle length (days)	31.0 ± 4.8	29.8 ± 1.9	23.2 ± 0.9	42.5 ± 6.8	<0.001
Period length (days)	5.7 ± 1.2	5.7 ± 1.2	5.4 ± 1.4	5.9 ± 1.2	<0.001
Admission BMI (kg/m^2^)	27.2 ± 3.4	27.1 ± 3.4	27.2 ± 3.3	27.6 ± 3.7	<0.001
Pre-pregnancy BMI (kg/m^2^)	22.2 ± 3.4	22.1 ± 3.3	22.1 ± 2.9	22.9 ± 3.9	<0.001
Gestational diabetes, n (%)	6,588 (37.0%)	5,714 (36.6%)	68 (33.8%)	750 (41.4%)	<0.001
Thyroid dysfunction, n (%)	5,803 (32.6%)	5,079 (32.5%)	66 (32.8%)	591 (32.7%)	0.991
ART conception, n (%)	600 (3.4%)	526 (3.4%)	10 (5.0%)	56 (3.1%)	0.365
Gestational hypertension, n (%)	1,333 (7.5%)	1,145 (7.3%)	12 (6.0%)	162 (9.0%)	0.033
Preterm birth, n (%)	1,089 (6.1%)	920 (5.9%)	17 (8.5%)	138 (7.6%)	0.005
Low birth weight, n (%)	880 (4.9%)	734 (4.7%)	12 (6.0%)	119 (6.6%)	0.002
Macrosomia, n (%)	691 (3.9%)	580 (3.7%)	9 (4.5%)	92 (5.1%)	0.015

Mean ± SD or n (%). Reference category: Normal cycle (25–35 days). GDM, gestational diabetes mellitus. TF, thyroid dysfunction (83.7% clinical hypothyroidism, 6.5% hyperthyroidism, 5.4% thyroiditis, 4.4% other). ART, assisted reproductive technology. P values: one-way ANOVA (continuous) and Pearson chi-square test (categorical), comparing the Normal, Short, and Long groups. Cycle length was missing for 168 women (0.9%), retained in the cohort total but excluded from cycle-length analyses (15,613 + 201 + 1,810 + 168 = 17,792; analytic cycle-length denominator = 17,624). Short-cycle estimates (n = 201, 1.1%) are exploratory. Repeat pregnancies: 152/17,792 (0.9%); the independence assumption was confirmed by GEE (sensitivity analysis SA1). The original GDM-stratified baseline table is provided as **Supplementary Table 9**.

**Figure 1:**
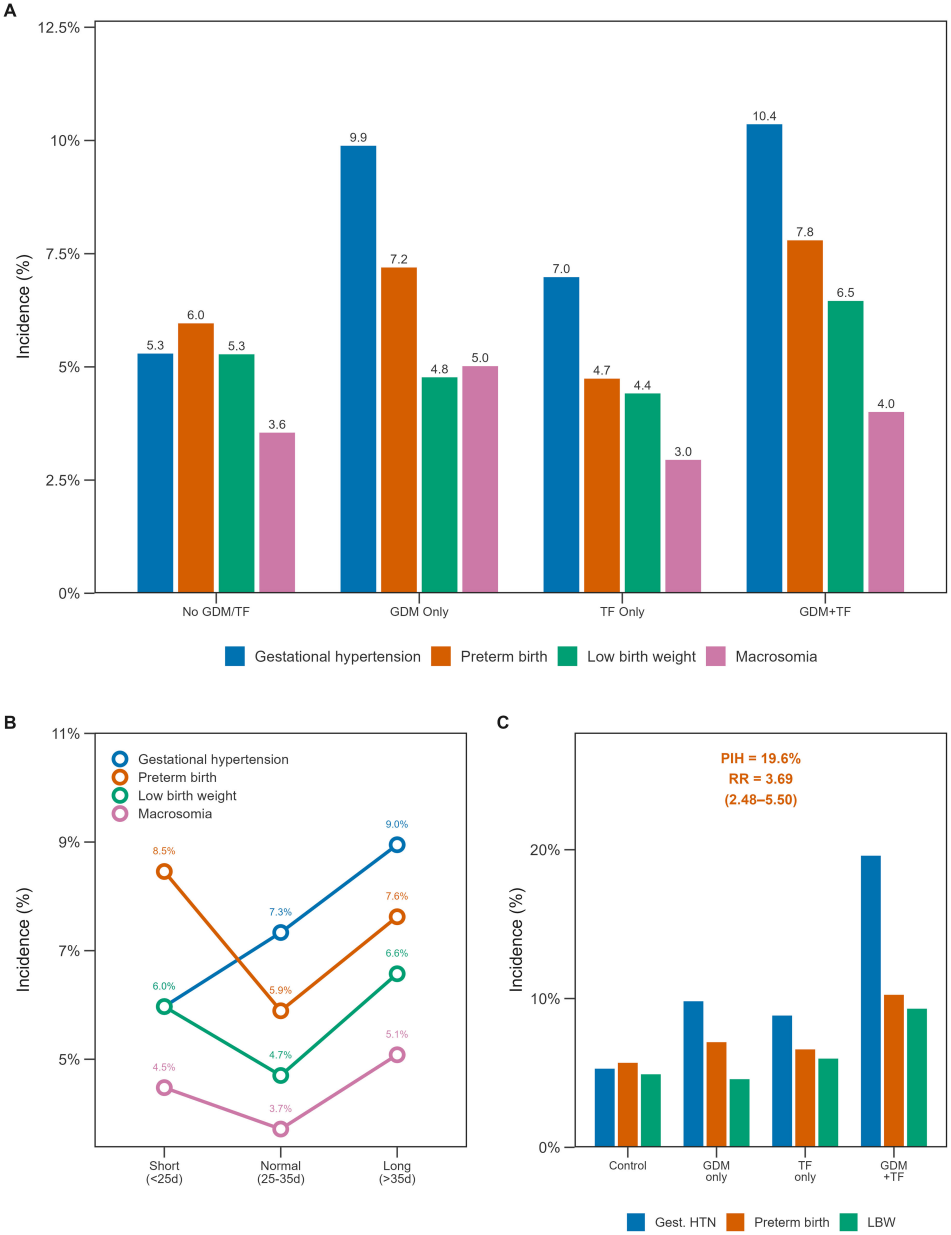
Descriptive overview: adverse outcome rates by metabolic subgroup and menstrual cycle category. **(A)** Incidence of gestational hypertension, PTB, LBW, and macrosomia by metabolic subgroup. Key observation: LBW rates are nearly identical between GDM and no-GDM groups (5.0% vs 4.9%; *p* = 0.8)—GDM-associated risk is concentrated in macrosomia, not LBW, indicating that LBW prediction may require risk markers beyond metabolic screening, such as cycle length. **(B)** Outcome rates by cycle category (Short/Normal/Long), showing monotonic increases in PTB and LBW with longer cycles. TF subtype: 83.7% clinical hypothyroidism. **(C)** Outcome rates in selected extreme-risk combinations: Control (no GDM/TF, normal cycle) vs GDM-only (normal cycle), TF-only (long cycle), and GDM+TF (long cycle). The GDM+TF+long-cycle subgroup had the highest gestational hypertension rate, illustrating the cumulative burden of metabolic and menstrual risk factors. Risk ratios are calculated relative to the Control group.

The GDM-stratified baseline comparison is provided in **Supplementary Table 9**. Thyroid dysfunction was more prevalent in the non-GDM group (4,906/11,204; 43.8%) than in the GDM group (897/6,588; 13.6%), indicating distinct rather than overlapping metabolic phenotypes (GDM×thyroid-dysfunction cross-tabulation, **Supplementary Table 10**; χ^2^ = 1717.1, *p* < 0.001). The cross-tabulation confirms that co-occurrence of GDM and thyroid dysfunction was lower than expected under independence (GDM + thyroid dysfunction 897/17,792, 5.0%), reflecting the two conditions’ distinct ascertainment pathways rather than a biological inverse relationship; annual prevalences and screening coverage are also reported in **Supplementary Table 10**; this distribution likely reflects the referral and diagnostic pathway structure of our tertiary center, where thyroid dysfunction—predominantly clinical hypothyroidism (83.7%)—is often identified through pre-conception screening, whereas GDM is diagnosed later in pregnancy (24–28 weeks) via universal OGTT. Notably, LBW prevalence did not differ by GDM status (No GDM 4.9% vs GDM 5.0%; p = 0.8; **Supplementary Table 9**), indicating that GDM-associated risk is concentrated in macrosomia, not LBW. Thus, LBW may be an outcome whose prediction could benefit from additional risk dimensions not captured by metabolic screening, such as cycle length (**Figure 1A**).

### Long cycle independently predicts PTB and LBW beyond GDM and thyroid dysfunction

**Figure 2:**
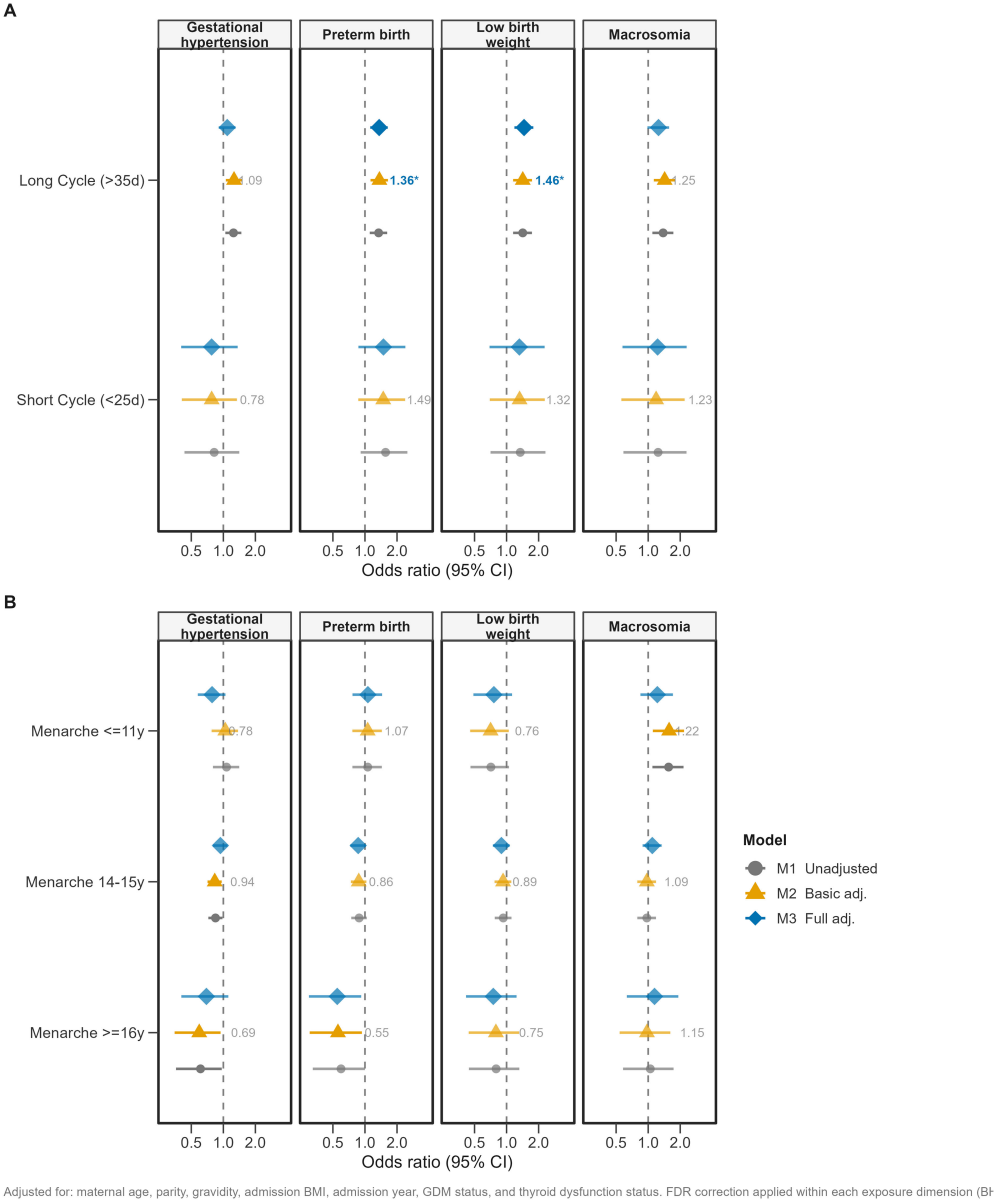
Forest plots of main effects across M1–M3. **(A)** Cycle length (reference: Normal 25–35d) vs four primary outcomes. Critical feature: ORs for long cycle → PTB (1.34/1.37/1.36) and → LBW (1.42/1.43/1.46) are near-identical across M1 to M3, demonstrating that GDM and thyroid dysfunction adjustment does not attenuate the associations—consistent with non-redundant risk information not materially explained by measured metabolic comorbidities. This stability primarily rules out GDM and TF as strong confounders, but does not establish causal independence. In contrast, gestational hypertension M1 OR (1.25) attenuates substantially in M3 (1.09, NS), indicating metabolic mediation of that association. **(B)** Menarche age vs four primary outcomes. Asterisk (*) = BH-FDR *p* < 0.05 in M3. M3: adjusted for age, parity, gravidity, BMI, year, GDM, TF. Short cycle (n = 201, 1.1%): substantially underpowered (*post-hoc* power <35% for OR = 1.36); results are exploratory only.

Long cycle (>35 days) was independently associated with PTB (aOR 1.36, 95% CI 1.12–1.64; BH-FDR *p* = 0.006) and LBW (aOR 1.46, 95% CI 1.18–1.78; BH-FDR *p* = 0.002) in the fully adjusted M3 model (**Table 2**; **Figure 2A**). On the absolute scale, these correspond to adjusted risk differences of +18.3 preterm births per 1,000 women (95% CI + 5.5 to +31.8; *p* = 0.012) and +19.2 low-birth-weight infants per 1,000 women (95% CI + 5.7 to +30.6; *p* < 0.001) for long-cycle compared with normal-cycle women, estimated by g-computation (marginal standardisation) from the M3 model (**Supplementary Table 11**). The defining feature of these associations is not their magnitude alone, but their stability across progressive covariate adjustment: ORs were 1.34 (M1), 1.37 (M2), and 1.36 (M3) for PTB, and 1.42, 1.43, and 1.46 for LBW. The near-identical M1 and M3 estimates demonstrate that neither GDM nor thyroid dysfunction accounts for the observed cycle-length associations – the metabolic comorbidities and menstrual cycle phenotype provide non-redundant risk information for PTB and LBW. This stability primarily rules out GDM and thyroid dysfunction as strong confounders and is consistent with (though does not prove) independent causal pathways: knowing a woman’s GDM status does not subsume her cycle-length-related PTB risk. In contrast, gestational hypertension showed a different pattern: the crude OR for long cycle (1.25) was substantially attenuated to 1.09 (not significant) in M3. This attenuation indicates that the apparent hypertension association was largely mediated or confounded by metabolic comorbidities, consistent with gestational hypertension being more directly tied to the metabolic pathway. Short cycle showed no FDR-significant association with any primary outcome. No menarche age category survived FDR correction after full adjustment (all FDR *p* > 0.43; **Figure 2B**). Spearman correlations between cycle length and primary outcomes were near zero (*ρ* = 0.00–0.02), consistent with a modest association detectable in regression but not in bivariate correlation of binary outcomes (**Supplementary Figure 7**).

**Table 2:** Main effects of menstrual phenotype dimensions on primary outcomes — M3 fully adjusted model.

Exposure	Outcome	aOR (95% CI)	*P* value	BH-FDR *P*	M1 OR†
Panel A. Cycle length (reference: Normal 25–35 days)					
Long (>35d)	Preterm birth	1.36 (1.12–1.64)	0.001	0.006**	1.34
Long (>35d)	Low birth weight	1.46 (1.18–1.78)	<0.001	0.002**	1.42
Long (>35d)	Gestational HTN	1.09 (0.90–1.30)	0.364	0.471	1.25‡
Long (>35d)	Macrosomia	1.25 (0.98–1.57)	0.061	0.164	1.38
Short (<25d)§	Preterm birth	1.49 (0.87–2.40)	0.120	0.241	1.56
Short (<25d)§	Low birth weight	1.32 (0.69–2.28)	0.358	0.471	1.34
Panel B. Menarche age (reference: 12–13 years)					
≤11 years	Macrosomia	1.22 (0.85–1.71)	0.268	0.448	1.56
≤11 years	Preterm birth	1.07 (0.76–1.45)	0.698	0.698	1.06
≥16 years	Macrosomia	1.15 (0.63–1.92)	0.627	0.684	1.05
Panel C. Period length (reference: Normal 3–7 days) — see Supplementary Table 23 for power analysis					
Long (>7d)¶	Preterm birth	1.04 (0.65–1.59)	0.854	0.997	1.00
Long (>7d)¶	Low birth weight	1.00 (0.59–1.59)	0.997	0.997	1.02
Long (>7d)¶	Gestational HTN	1.58 (1.06–2.27)	0.024	0.171	1.58

aOR, adjusted odds ratio from M3 (adjusted for: maternal age, parity, gravidity, admission BMI, admission year, GDM status, thyroid dysfunction status). BH-FDR, Benjamini–Hochberg correction within each exposure dimension. **FDR *p* < 0.01, *FDR *p* < 0.05. †M1 (crude) OR shown to illustrate stability across covariate adjustment. ‡Gestational HTN, substantial M1→M3 attenuation (1.25→1.09) indicates metabolic mediation. §Short cycle (n = 201, 1.1%): substantially underpowered; exploratory only. ¶Long period (n=339 total, 1.9%; n=335 complete-case analysis): power <50% for OR ≤1.36 (**Supplementary Table 1**); null result is directional evidence only.

### Monotonic dose–response across the cycle-length spectrum

**Figure 3:**
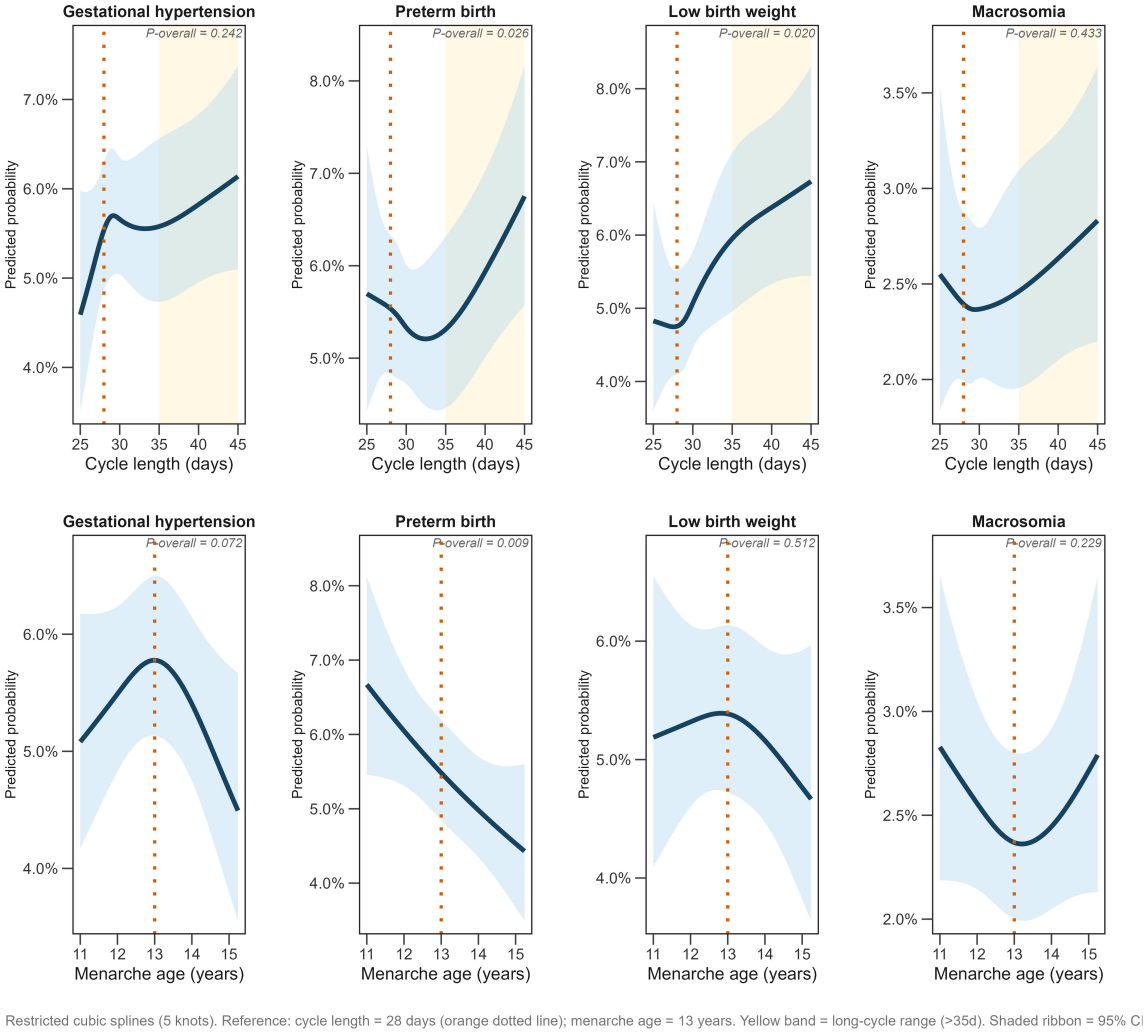
RCS dose–response: continuous cycle length vs predicted probability of PTB and LBW (M3). Reference: 28 days. Shaded band: 95% CI. *P*-overall = 0.026 (PTB) and 0.020 (LBW). Risk increases monotonically from approximately 30 days; no inflection at 45 days or longer (as would be expected if a discrete PCOS subgroup were driving results), providing indirect evidence against undiagnosed PCOS confounding. P-nonlinear > 0.20 for both outcomes; linear trend predominates. No significant dose–response for gestational hypertension or macrosomia.

RCS analysis confirmed a significant monotonic positive association between continuous cycle length and both PTB (*P*-overall = 0.026; *P*-nonlinear = 0.204) and LBW (*P*-overall = 0.020; *P*-nonlinear = 0.312) (**Figure 3**). Risk estimates began to increase from approximately 28–30 days, before the conventional 35-day categorical threshold, suggesting a continuous dose–response across the cycle-length spectrum rather than a threshold effect at any single cut-point. Notably, no inflection was observed at ≥45 days, the range where a discrete PCOS subgroup effect would be expected if undiagnosed PCOS were driving the association. This monotonic, predominantly linear dose–response pattern argues against a threshold-driven effect expected from a discrete subgroup and provides indirect evidence against confounding by undiagnosed PCOS. No significant dose–response was detected for gestational hypertension or macrosomia.

### Menstrual and metabolic phenotypes provide independent, non-overlapping risk information

**Figure 4:**
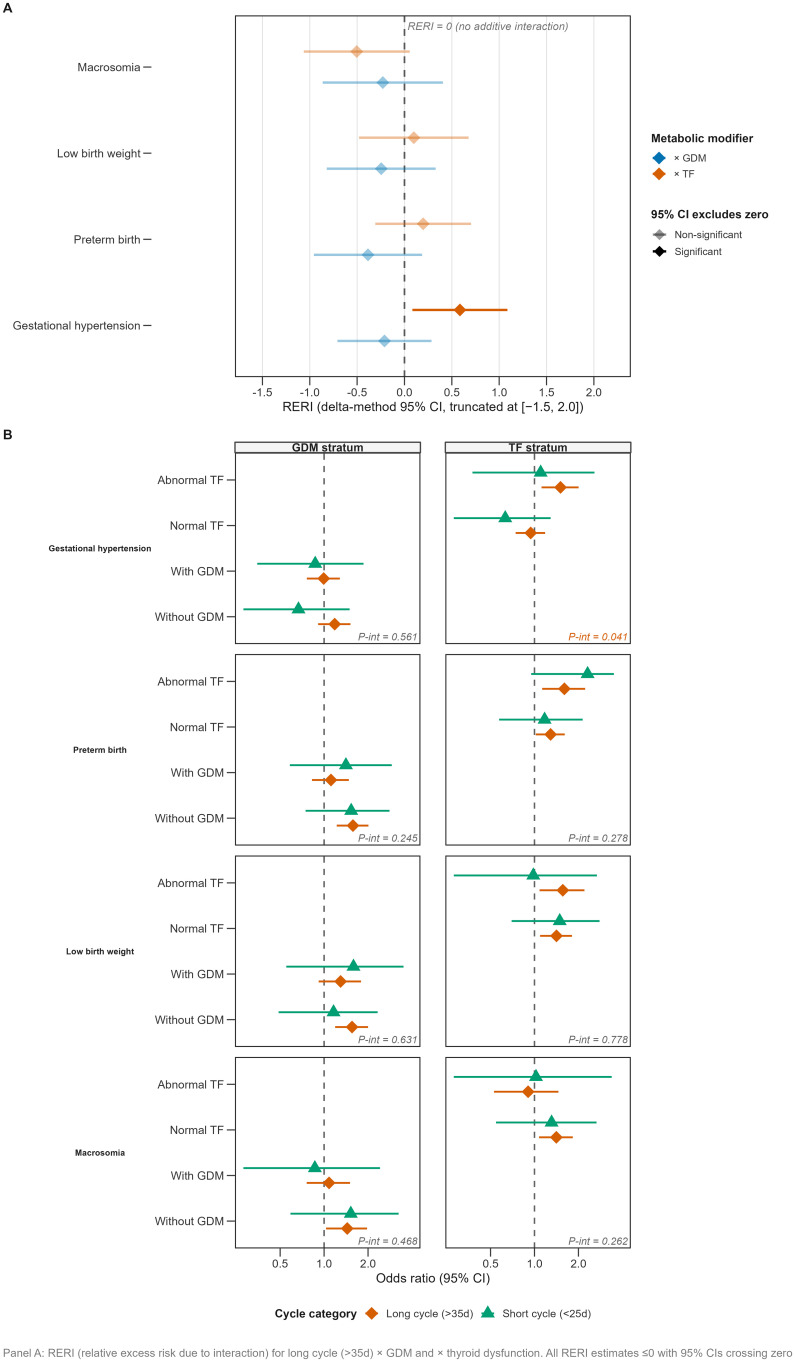
Parallel risk pathway evidence. **(A)** RERI forest plot: long cycle × GDM and × TF for four primary outcomes. All RERI estimates are ≤0 with 95% CIs crossing zero for GDM interactions, consistent with sub-additive or null interaction – no evidence of superadditive synergism. Absence of additive interaction does not prove causal independence, but indicates that combined risk does not exceed the sum of independent contributions. Delta-method CIs; interactionR package. **(B)** Stratified analysis: cycle-length ORs within GDM and TF strata. Consistent ORs across all metabolic strata confirm that the long-cycle → PTB/LBW association is present regardless of GDM or TF status (*P*-interaction >0.05 for all PTB and LBW combinations)—the risk information carried by cycle length is present in every metabolic stratum and is not subsumed by metabolic comorbidity status.

The RERI pattern provides supportive but not conclusive evidence for independent risk dimensions (**Table 3**; **Figure 4**; **Supplementary Figure 8**; **Supplementary Table 12** for full decomposition). For long cycle × GDM, all four RERI estimates were ≤0 (gestational hypertension: −0.21; PTB: −0.39; LBW: −0.25; macrosomia: −0.23), all with 95% CIs crossing zero, consistent with sub-additive or null interaction, providing no evidence of superadditive synergism on the additive scale. For long cycle × TF, RERI estimates for the two primary outcomes were small and positive but non-significant, with CIs crossing zero (PTB × TF: RERI + 0.20, 95% CI −0.31 to +0.70; LBW × TF: RERI + 0.10, 95% CI −0.48 to +0.68), providing no evidence of superadditive synergism, though statistical power to detect moderate interaction was limited. CI upper bounds for the primary GDM interactions excluded clinically meaningful synergism (PTB × GDM upper CI + 0.19, corresponding to less than 1 percentage point absolute excess risk above additive prediction; LBW × GDM upper CI + 0.33). Absence of additive interaction should not be interpreted as proof of independent causal pathways, but rather as lack of superadditive amplification, consistent with cycle-length risk being present across metabolic strata rather than concentrated in any single subgroup. The clinical implication for the primary outcomes is that the additional risk statistically associated with long cycle (PTB PAF 3.1%, LBW PAF 3.9%) remains accessible only through explicit menstrual history inquiry; it cannot be inferred from metabolic screening results. For multiplicative interaction (**Supplementary Table 13**), LRT P-values were >0.08 for all PTB and LBW combinations. Stratified analyses confirmed consistent long-cycle ORs across all GDM and thyroid strata (**Figure 4B**), with no significant effect modification, demonstrating that the risk information carried by cycle length is non-redundant with metabolic comorbidity information in every metabolic stratum.

**Table 3:** Additive interaction (RERI, AP, SI): Long cycle × GDM and Long cycle × TF.

Outcome	Interaction	OR_E1†	OR_E2†	OR_both†	RERI	AP	SI
Gest. HTN	Long × GDM	1.20 (0.93–1.54)	1.52 (1.34–1.74)	1.52 (1.16–1.96)	−0.211 (−0.708, 0.285)	−0.139 (−0.490, 0.211)	0.710 (0.308–1.638)
PTB	Long × GDM	1.55 (1.20–1.98)	1.33 (1.15–1.53)	1.49 (1.11–1.98)	−0.385 (−0.957, 0.186)	−0.258 (−0.689, 0.173)	0.562 (0.221–1.427)
LBW	Long × GDM	1.54 (1.18–1.99)	1.01 (0.86–1.19)	1.31 (0.93–1.80)	−0.246 (−0.821, 0.329)	−0.187 (−0.666, 0.291)	0.559 (0.127–2.463)
Macrosomia	Long × GDM	1.37 (0.98–1.88)	1.24 (1.04–1.48)	1.39 (0.98–1.92)	−0.229 (−0.864, 0.407)	−0.165 (−0.656, 0.327)	0.629 (0.165–2.396)
Gest. HTN	Long × TF	0.93 (0.73–1.17)	1.10 (0.96–1.27)	1.62 (1.21–2.14)	+0.586 (0.084, 1.087)	+0.362 (0.130, 0.594)	19.198 (0.003–125911.867)
PTB	Long × TF	1.27 (1.00–1.58)	0.85 (0.72–0.99)	1.31 (0.93–1.79)	+0.197 (−0.308, 0.703)	+0.151 (−0.200, 0.502)	2.767 (0.119–64.308)
LBW	Long × TF	1.39 (1.08–1.78)	0.90 (0.76–1.07)	1.40 (0.98–1.94)	+0.099 (−0.479, 0.677)	+0.071 (−0.326, 0.467)	1.332 (0.249–7.109)
Macrosomia	Long × TF	1.39 (1.06–1.80)	0.94 (0.77–1.14)	0.82 (0.48–1.30)	−0.504 (−1.063, 0.055)	−0.615 (−1.518, 0.288)	NE

RERI, relative excess risk due to interaction; AP, attributable proportion; SI, synergy index. RERI>0, AP>0, SI>1 = superadditive synergism. †Component ORs derived by joint 4-level logistic regression (Neither/Long-cycle-only/Modifier-only/Both), fully adjusted (M3). RERI_implicit = OR_both − OR_E1 − OR_E2 + 1 (**Supplementary Table 12** for full decomposition). Delta-method 95% CI (interactionR). Shaded rows = TF interaction. GDM models adjusted for TF; TF models adjusted for GDM. Key: for long cycle × GDM, all four RERI ≤0 (range −0.39 to −0.21); for long cycle × TF, primary outcome CIs cross zero. No superadditive interaction for PTB or LBW. ††The extremely wide SI confidence interval for Gest. HTN × TF (0.003–125,911.867) reflects numerical instability when component ORs are near 1.0; SI is uninformative in this row and RERI should be used as the primary interaction metric.

### Birth weight reduction reflects both earlier delivery and impaired fetal growth

**Figure 5:**
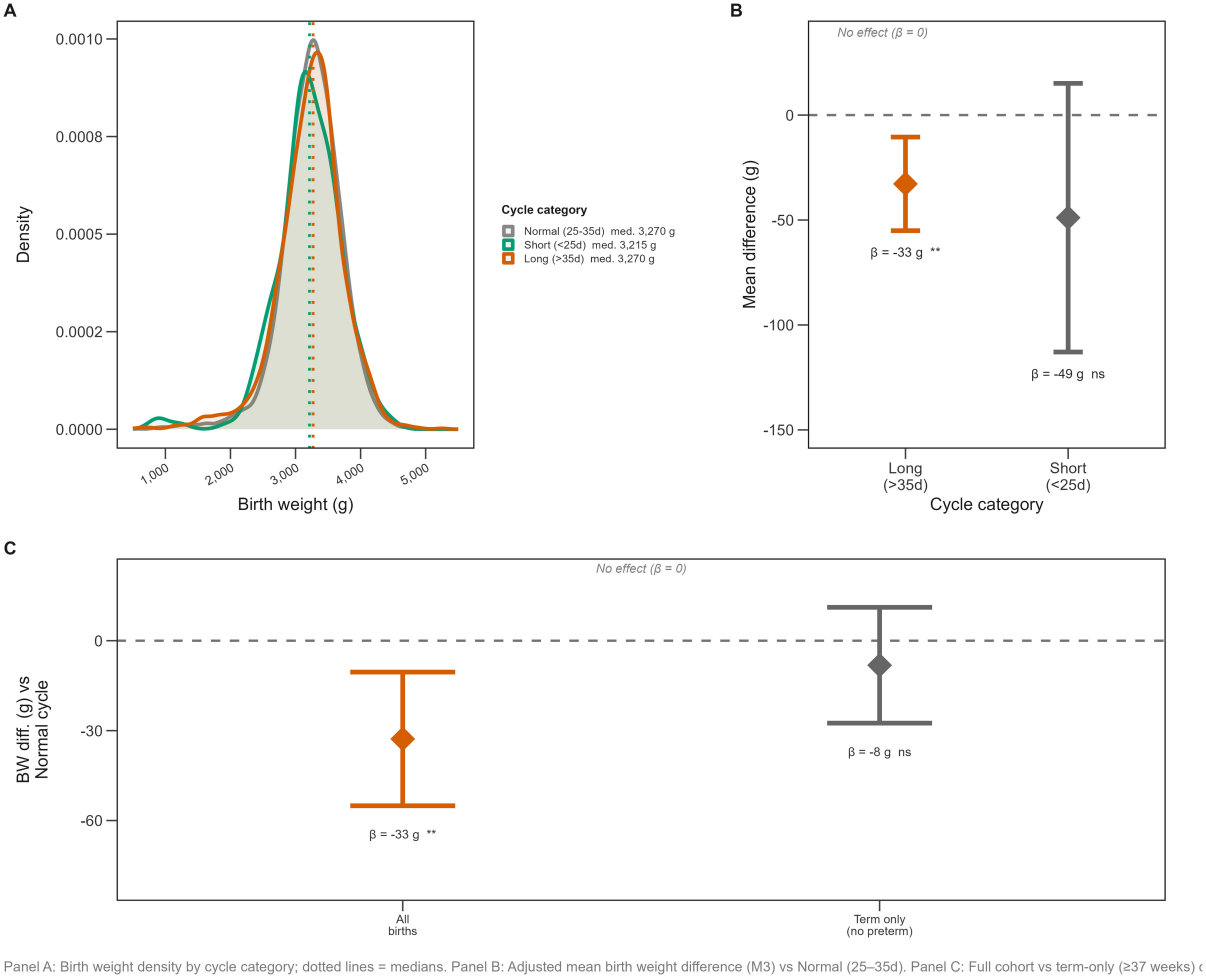
Birth weight analysis and PTB-mediation evidence. **(A)** Birth weight density by cycle category; medians: Short 3,215 g, Normal 3,270 g, Long 3,270 (g) **(B)** Adjusted birth weight difference: full cohort *β* = −33 g (*p* < 0.01) for long vs normal cycle. **(C)** Term deliveries only (≥37 weeks): *β* = −8 g (not significant). The near-complete attenuation in the term-only analysis is consistent with the birth weight signal being largely PTB-mediated (delivery timing); however, a size-for-gestational-age analysis (**Supplementary Table 14**) shows long cycle is also associated with SGA, indicating an additional, smaller growth-restrictive component. Formal causal mediation analysis was not performed. M3 adjusted.

In the full cohort, long cycle was associated with a mean birth weight reduction of 33 g (*β* = −33 g, *p* < 0.01) (**Figure 5**). Among term deliveries only (≥37 weeks), the birth weight difference was −8 g (not significant), with nearly identical medians across cycle groups (Short 3,215 g; Normal 3,270 g; Long 3,270 g). The near-complete attenuation of the birth weight deficit in the term-only analysis is consistent with the birth weight signal being mediated by preterm birth (reflecting delivery timing) partly, although a size-for-gestational-age analysis (below) indicates that impaired fetal growth also contributes. This distinction is clinically important: it suggests that the effect of long cycle is predominantly on the timing of delivery, with an additional, smaller effect on fetal growth. In a *post-hoc* exploratory analysis, we classified preterm births as spontaneous (preterm premature rupture of membranes or spontaneous labour onset; n = 773, 71.0%), indicated (delivery for documented maternal/fetal indications; n = 128, 11.8%), or uncertain (n = 188, 17.3%) using ICD-10-coded EHR diagnoses. In fully adjusted models the long-cycle association was significant for spontaneous PTB (aOR 1.30, 95% CI 1.04–1.62; *p* = 0.020) but not for indicated PTB (aOR 1.22, 95% CI 0.68–2.05; *p* = 0.468; 122 events), the latter substantially underpowered (**Supplementary Table 2**). Gestational-age subtypes [early (<34 weeks) vs late (34–36 weeks) PTB] could not be reliably analysed owing to small early-PTB event counts.

To distinguish impaired fetal growth from earlier delivery, we examined size-for-gestational-age using INTERGROWTH-21st birthweight z-scores (n = 17,303 evaluable; mean z +0.18, SD 0.88; SGA 4.5%, LGA 10.3%, consistent with previously reported higher birthweights in Chinese populations relative to the international standard). Long cycle was associated with higher odds of SGA (aOR 1.39, 95% CI 1.11–1.73; *p* = 0.003) and with a small reduction in birthweight z-score (β −0.052, 95% CI −0.093 to −0.010; *p* = 0.015) after full adjustment. The association with SGA persisted among term deliveries (≥37 weeks: aOR 1.34, 95% CI 1.07–1.69; *p* = 0.013), whereas the continuous z-score reduction attenuated and was no longer significant at term (*β* −0.037; *p* = 0.089); the estimate for severe SGA/FGR (<3rd centile) was directionally consistent but underpowered (164 events; aOR 1.40, 95% CI 0.89–2.20). Together these findings indicate that the birthweight signal associated with long cycle reflects both earlier delivery and a component of impaired fetal growth, rather than delivery timing alone (**Supplementary Table 14**).

### Period length: null associations consistent with but not establishing dimension specificity

A long period (>7 days; n = 339, 1.9%) showed no association with PTB (aOR 1.04, 95% CI 0.65–1.59; *p* = 0.854; FDR *p* = 0.997) or LBW (aOR 1.00; *p* = 0.997) (**Supplementary Figure 9**; **Table 2** Panel C). The PAF for long period was 0.0% for PTB (95% CI −0.8% to 0.8%) and 0.0% for LBW – approximately 31-fold lower than for long cycle (**Supplementary Figure 10**). If the association with PTB and LBW were a general feature of menstrual health disruption, both cycle-length and period-length abnormalities would be expected to carry comparable risk. The concentrated attribution in the cycle-length (ovulatory interval) dimension, with near-zero attribution in the period-length (endometrial) dimension, is directionally consistent with the effect being more specific to ovulatory timing than to menstrual health in general. The null period-length findings are more accurately characterized as consistent with dimension specificity rather than as evidence for it. With *post-hoc* power of 7–35% for ORs of 1.10–1.36 (n = 335 exposed in complete-case analysis; **Supplementary Table 1**), this comparison cannot distinguish a true null effect from an effect of similar magnitude to that observed for cycle length. What can be stated is that the data contain no signal from period length for either PTB or LBW – a finding directionally consistent with the ovulatory interval hypothesis, but one that should be explicitly framed as generating rather than testing that hypothesis. The comparison is better viewed as a pre-specified exploratory dimension analysis than as a controlled internal negative control; the term “dimension-specificity comparison” reflects its design intent, not its evidential force. An adequately powered test would require approximately 1,200–1,500 exposed long-period women, achievable in a multi-center prospective study that pre-specifies period length as a co-primary exposure. The null period-length findings should not be interpreted as excluding the possibility of a modest period-length association with PTB or LBW; they are underpowered (**Supplementary Table 1**) and pending an adequately powered replication study. Additive interaction analyses for long period × GDM and × TF, PAF estimates for period length, and sensitivity analyses restricting to pre-pregnancy BMI and thyroid-dysfunction-free subsets are presented in **Supplementary Tables 15**, **S16**, and **S17**, respectively; all findings were directionally consistent with the primary period-length null result. *Post-hoc* power analysis documented that the period-length comparison was substantially underpowered (n=335 exposed; power 7.1–35.4% for OR 1.10–1.36; **Supplementary Table 1**). The null result for period length and the PAF contrast are directional evidence only – they are hypothesis-generating and cannot constitute proof of dimension specificity without an adequately powered replication study (requiring approximately 1,200–1,500 exposed long-period women for 80% power at OR = 1.36).

### Secondary outcomes and null findings

Secondary outcomes – amniotic fluid abnormality and fetal distress – showed no significant associations with menstrual cycle length category after FDR correction (all FDR *p* > 0.93; **Supplementary Table 18**). These outcomes were therefore not analyzed further. Notably, 75.2% of fetal distress cases co-occurred with amniotic fluid abnormality, consistent with the expectation that these outcomes share a common obstetric pathway and are not independently informative in this context.

### Population-attributable fractions, E-values, and sensitivity analyses

PAF attributable to long cycle: PTB 3.1% (95% CI 0.9–5.4%) and LBW 3.9% (1.2–6.4%), with bootstrap CIs excluding zero (**Supplementary Table 19**). In this tertiary referral cohort, where GDM prevalence was 37.0% and thyroid dysfunction prevalence was 32.6%, these PAF estimates quantify the proportion of outcome occurrence statistically associated with long cycle under model assumptions and should not be interpreted as directly reflecting a causal attributable burden. Because long cycle and metabolic comorbidities are non-synergistic (RERI ≈ 0), the cycle-length-attributable fraction is independent of the metabolic-comorbidity-attributable fraction in this population. These PAF values are specific to this tertiary referral setting and are not directly generalizable to community obstetric populations. PTB E = 2.06 (CI lower bound 1.49); LBW E = 2.28 (CI lower bound 1.64) (**Supplementary Table 20**; **Supplementary Figure 11**). An unmeasured confounder would require risk ratio associations exceeding these thresholds with both exposure and outcome to fully explain the observed associations. Results were consistent across all five sensitivity analyses (PTB aOR range 1.27–1.41; LBW 1.40–1.42), confirming robustness to BMI ascertainment method, thyroid status inclusion, ART exclusion, and subtype restriction. In a further sensitivity analysis addressing maternal infection — a recognised cause of preterm birth — infection-related diagnoses (chorioamnionitis, urinary tract infection, and genital-tract and other maternal infections; overall prevalence 11.3%, and modestly higher in the long-cycle group [13.1%] than the normal-cycle group [11.1%]) were added to the M3 model. The long-cycle estimates were essentially unchanged (PTB aOR 1.36→1.35; LBW aOR 1.46→1.45; **Supplementary Table 21**), indicating that the cycle-length associations are not materially confounded by documented infection. Incremental discriminative analysis showed that menstrual cycle length provides statistically significant but numerically modest improvement in predicted probabilities beyond the base metabolic model. Adding cycle category yielded ΔAUC = 0.0055 for PTB (DeLong *p* = 0.131) and ΔAUC = 0.0071 for LBW (DeLong *p* = 0.146). However, the Integrated Discrimination Improvement was statistically significant for both outcomes: IDI = 0.00069 (95% CI 0.00022–0.00118; *p* = 0.002) for PTB and IDI = 0.00088 (95% CI 0.00038–0.00139; *p* < 0.001) for LBW, indicating that the full model systematically assigns higher predicted probabilities to women who subsequently experienced these outcomes. Continuous NRI was 0.0700 (95% CI 0.0269–0.1134; *p* < 0.001) for PTB and 0.0785 (95% CI 0.0315–0.1292; *p* = 0.002) for LBW. The primary value of menstrual cycle length is in providing non-redundant risk information not captured by metabolic screening, as quantified by the statistically significant IDI and population-attributable fractions, rather than in large standalone discriminative impact (full results in **Supplementary Table 22**; **Supplementary Figure 12**). Both models were well calibrated across the range of predicted risk, with decile-level observed rates closely tracking the line of perfect calibration and no systematic over- or under-prediction after adding cycle category (**Supplementary Figure 5**). On decision-curve analysis, the full model yielded a small positive increment in net benefit over the base model across low threshold probabilities (approximately 0–15%), the range relevant to preterm-birth and low-birth-weight screening, and no loss of net benefit elsewhere (**Supplementary Figure 6**). Consistent with the modest discrimination metrics, the magnitude of the net-benefit gain was small; these analyses indicate that adding cycle category does not degrade calibration and confers a small clinical-utility increment rather than a large one.

### Residual PCOS confounding: quantitative bias analysis

**Figure 6:**
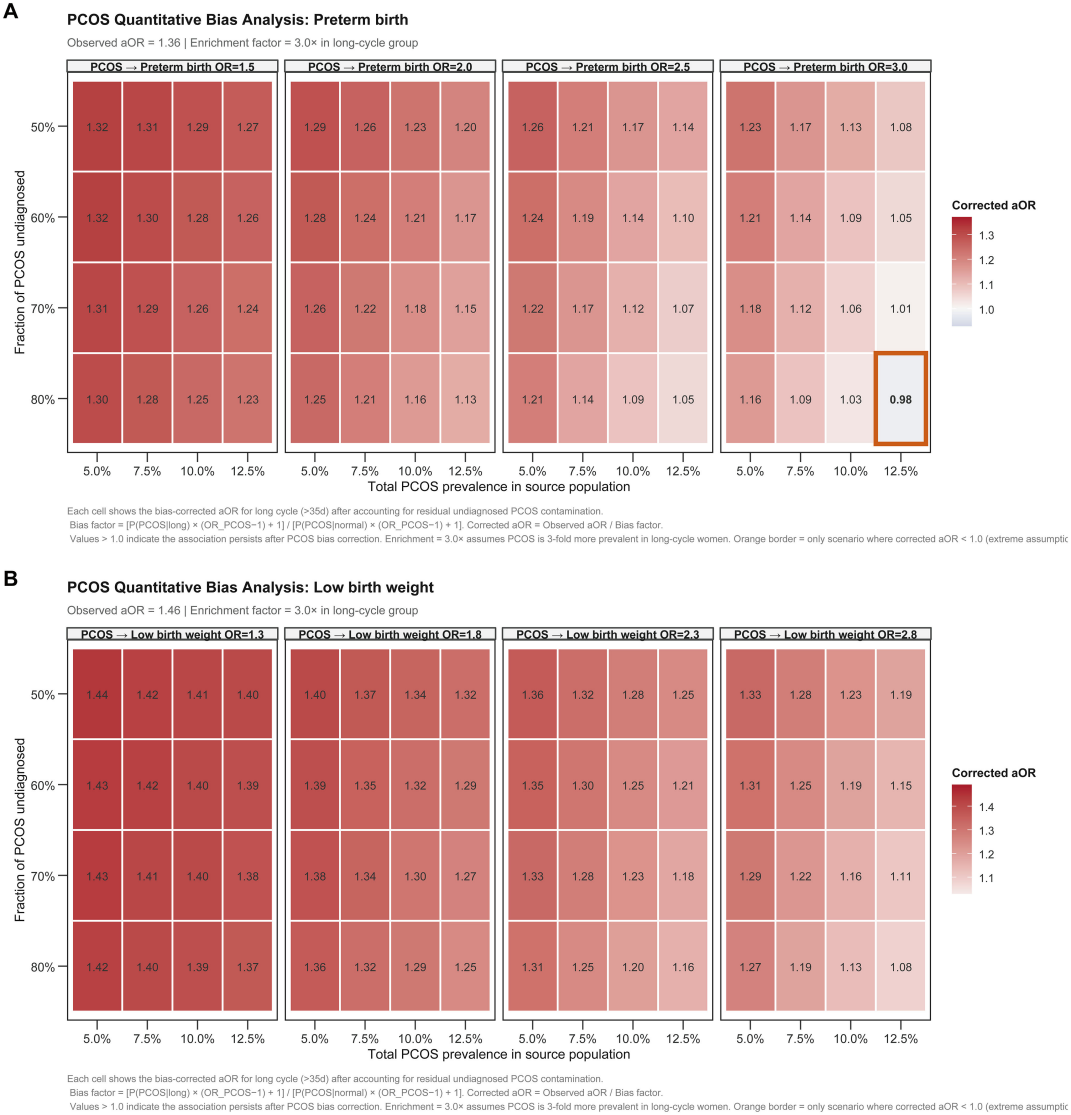
PCOS quantitative bias analysis (QBA): sensitivity of long-cycle aOR to residual undiagnosed PCOS confounding after four-source clinical exclusion. **(A)** Preterm birth. Observed aOR = 1.36; enrichment factor = 3× in the long-cycle group (conservative post-exclusion assumption; see text and Results). **(B)** Low birth weight. Observed aOR = 1.46; enrichment factor = 3×. Each panel is a 4×4 heat map grid of bias-corrected aOR across 16 parameter combinations: total PCOS prevalence in the source population (5.0%, 7.5%, 10.0%, 12.5%) × fraction undiagnosed (50%, 60%, 70%, 80%), evaluated under four PCOS→outcome OR assumptions (1.5, 2.0, 2.5, 3.0). The 3× enrichment assumption is conservative: four-source clinical exclusion (n = 245 women removed) preferentially depleted Rotterdam Phenotypes A–C from the long-cycle group before analysis, reducing residual enrichment well below the 7–8-fold biological ceiling of unselected populations. Under this assumption, 63/64 PTB scenarios and 64/64 LBW scenarios retain bias-corrected aOR >1.0 (green shading). Under 2× enrichment, all 64/64 scenarios for both outcomes retain corrected aOR >1.0; under the more stringent 5× assumption, 43/64 PTB and 52/64 LBW scenarios retain corrected aOR >1.0. The monotonic RCS dose–response without inflection at ≥45 days (**Figure 3**) provides additional indirect evidence against confounding by a discrete PCOS subgroup. Full numerical grid in **Supplementary Table 24**.

Because long menstrual cycles (>35 days) share biological overlap with PCOS-associated oligomenorrhea, we conducted a formal quantitative bias analysis (QBA) to assess whether residual undiagnosed PCOS could account for the observed associations. All clinically recognized PCOS cases (n = 245) were excluded using a four-source ascertainment approach (ICD-10 coding, clinician diagnosis, patient self-report, and structured clinical note review). Before exclusion, PCOS prevalence rose steeply across cycle-length categories — 0.91% in short-cycle, 0.50% in normal-cycle, and 7.1% in long-cycle women (χ^2^ = 653, *p* < 0.001; **Supplementary Table 23**), a 14-fold enrichment in the long-cycle group. Because 64% of all PCOS cases occurred among long-cycle women, the four-source exclusion preferentially removed PCOS from precisely the stratum most relevant to the exposure, supporting the subsequent quantitative bias analysis of residual undiagnosed PCOS. Because oligomenorrhea characterizes approximately 75–85% of women with PCOS, this four-source exclusion preferentially depleted the long-cycle group of its highest PCOS concentration stratum before analysis, reducing residual enrichment well below the 7–8-fold biological ceiling implied by oligomenorrhea prevalence data in unselected populations. We applied a conservative 3× post-exclusion enrichment assumption across a 64-scenario parameter grid spanning PCOS prevalence (5.0–12.5%), undiagnosed fraction (50–80%), and PCOS→outcome OR (1.5–3.0) (**Figure 6**; **Supplementary Table 24**). Under this conservative assumption, 63 of 64 PTB scenarios and all 64 LBW scenarios retained bias-corrected aOR >1.0; under the less extreme 2× enrichment assumption, all 64 scenarios for both outcomes retained corrected aORs >1.0. The monotonic RCS dose–response without inflection at ≥45 days – the range where a discrete PCOS subgroup effect would be expected – provides additional indirect evidence against confounding by a residual undiagnosed PCOS subgroup (**Figure 3**). Taken together, these analyses indicate that the observed associations between long cycle length and PTB/LBW are robust to plausible levels of residual PCOS confounding following four-source clinical exclusion.

## Discussion

In this large real-world cohort of 17,792 deliveries from a Chinese tertiary center with a high metabolic comorbidity burden, we show that menstrual cycle length – specifically prolonged cycles (>35 days)—is independently associated with increased risks of preterm birth and low birth weight, even after comprehensive simultaneous adjustment for gestational diabetes and thyroid dysfunction. Notably, the stability of effect estimates across progressively adjusted models (PTB: 1.34 to 1.36; LBW: 1.42 to 1.46), along with the absence of superadditive interaction (RERI ≈ 0), indicates that menstrual cycle length provides risk information not materially explained by measured GDM or TF – consistent with, though not proof of, cycle length and metabolic comorbidities carrying partially non-overlapping prognostic information.

Furthermore, the null findings for period length across all analyses, along with the monotonic dose–response observed for cycle length in RCS analyses, provide supportive but underpowered evidence for dimension-specificity, suggesting that the observed associations are more specific to the ovulatory interval than to menstrual characteristics in general. However, this evidence is directional rather than definitive, given the limited statistical power of the long-period group (n = 335 complete cases; power 7–35% for OR 1.10–1.36). Taken together, these findings support the interpretation that menstrual cycle length captures risk information for PTB and LBW that is not addressed by current antenatal metabolic screening frameworks. Our findings do not, however, redefine the biological architecture of perinatal risk or establish a distinct non-metabolic causal axis.

Current antenatal risk assessment is largely organized around metabolic disorders, particularly gestational diabetes and thyroid dysfunction, both of which are routinely screened for and intensively managed. Our findings suggest that this paradigm may be incomplete. Even in a setting with high prevalence and systematic screening of metabolic comorbidities, menstrual cycle length retained independent associations with adverse perinatal outcomes, without evidence of synergistic amplification. This pattern implies that menstrual cycle length is unlikely to be fully explained by measured metabolic dysfunction and may reflect additional, partially distinct biological processes, potentially involving hypothalamic–pituitary–ovarian axis regulation, luteal phase adequacy, or progesterone dynamics. However, we caution that our estimates should be interpreted as conditional associations rather than total causal effects, given the complex causal position of BMI – which may act as a mediator (if cycle length influences gestational weight gain) or a collider (if unmeasured factors jointly affect weight gain and preterm birth)—and which was only partially addressed by sensitivity analyses (see **Supplementary Figure 2**; **Supplementary Table 4**). The most biologically plausible candidate mechanism is luteal-phase insufficiency and relative progesterone deficiency in women with chronic anovulatory cycles (31), though the independent clinical relevance of luteal phase deficiency as a diagnostic entity remains debated. Progesterone supports myometrial quiescence and cervical competence during pregnancy (32–34), and vaginal progesterone supplementation reduces preterm birth in women with a sonographically short cervix (PREGNANT trial; RR 0.55) (22). The observed pattern – effect concentrated in cycle length but not period length, an association statistically significant for spontaneous but not indicated preterm birth (the latter *post-hoc* and underpowered), no PCOS-type inflection in the restricted cubic spline at ≥45 days, and a birth weight reduction that is mediated predominantly by preterm birth but with an additional small growth-restrictive component (long cycle was associated with SGA, including at term; **Supplementary Table 14**) – is collectively consistent with, but not specific to, an ovulatory-timing–related mechanism operating independently of hyperandrogenic pathways. A formal quantitative bias analysis confirmed robustness to plausible residual undiagnosed PCOS confounding (63/64 preterm birth and 64/64 low birth weight scenarios retained bias-corrected aOR >1.0; **Figure 6**; **Supplementary Table 24**). Mechanistically, chronic oligomenorrhoea reflects infrequent ovulation and abbreviated luteal phases, reducing cumulative progesterone exposure; women with oligomenorrhoeic cycles demonstrate flatter luteal progesterone profiles consistent with hypothalamic–pituitary–ovarian axis dysregulation upstream of gestational diabetes–associated metabolic manifestations (35). This pathway – oligomenorrhoea → luteal insufficiency → suboptimal progesterone milieu → preterm birth susceptibility – is mechanistically distinct from the insulin-resistance pathway linking long cycles to gestational diabetes, providing a biologically plausible explanation for why the two risk dimensions are non-redundant. We emphasize, however, that this account remains hypothetical because the hormonal measurements needed to test it directly were not available in the present dataset; the luteal-phase-insufficiency pathway is therefore inferred from the cycle-length phenotype and prior physiological work rather than demonstrated in this cohort. Prospective studies incorporating serial LH, FSH, and mid-luteal progesterone measurements are required to test this pathway directly.

We note an important methodological caveat regarding OR stability: the modest overlap between long cycle and GDM (11.4% vs 9.5% long-cycle prevalence) means that adjusting for GDM would not be expected to substantially attenuate the cycle-length OR, regardless of whether the underlying mechanisms are truly independent. OR stability across M1–M3 is therefore consistent with, but does not prove, independent causal pathways; it primarily rules out GDM and TF as strong confounders of the observed associations. While OR stability alone cannot establish causal independence, the convergence of multiple complementary analytical strands – stable RERI ≈ 0 across all primary outcome–modifier combinations, monotonic RCS dose–response without subgroup inflection, null period-length associations directionally consistent with ovulatory-interval specificity, QBA robustness across 64 PCOS confounding scenarios, and significant IDI/NRI – collectively makes a purely metabolic explanation unlikely to fully account for the observed associations, even though a causal pathway distinct from metabolic dysfunction cannot be established from observational data alone. Our findings build on the Project Viva cohort (Soria-Contreras et al., 2022; n = 2,046; RR 2.04 for PTB with long/irregular cycles) (23) by addressing the key unanswered question in that study: whether the association survives simultaneous adjustment for GDM and thyroid dysfunction in a high-burden population. It does, with an aOR of 1.36—somewhat lower than the Project Viva RR of 2.04, consistent with the substantial metabolic comorbidity burden in our cohort partially absorbing shared variance. An independent Australian longitudinal cohort (n = 6,615 mothers; 12,337 singleton births) found that pre-pregnancy menstrual irregularity, severe period pain, and heavy bleeding before a second or subsequent birth were each prospectively associated with elevated preterm birth risk (36), corroborating menstrual phenotype as a reproducible perinatal risk indicator across distinct population settings.

The RERI structure requires careful interpretation, with appropriate attention to statistical power. For long cycle × GDM, all four RERI point estimates were negative (range −0.21 to −0.39), with confidence intervals crossing zero, consistent with sub-additive or null interaction. For long cycle × TF, three of four estimates were positive (PTB: +0.20; LBW: +0.10; gestational hypertension: +0.59), though only the gestational hypertension interaction had a confidence interval excluding zero. The divergence between GDM and TF modifiers reflects a meaningful biological difference: GDM is characterized by insulin resistance, in which long cycle and GDM may share overlapping metabolic pathways, while TF – predominantly clinical hypothyroidism in this cohort – represents a distinct hormonal disruption with different mechanistic relationships to cycle length (37). A large-scale meta-analysis of 47,045 pregnancies confirmed that subclinical hypothyroidism, isolated hypothyroxinemia, and thyroid peroxidase antibody positivity each independently increase preterm birth risk (34); mechanistic reviews suggest these associations operate via immunological and thyroid hormone–mediated pathways distinct from insulin resistance (38, 39).

For the primary outcomes of greatest clinical interest – PTB and LBW – no RERI estimate had a 95% confidence interval entirely above zero, confirming the absence of superadditive synergism regardless of metabolic modifier. Confidence interval upper bounds for the primary GDM interactions excluded clinically meaningful synergism (PTB × GDM upper CI + 0.19; LBW × GDM upper CI + 0.33). We emphasize that statistical power to detect moderate superadditive interaction was limited (approximately 18–50% for RERI = 0.3–0.5; **Supplementary Table 8**), and the null RERI findings should therefore be interpreted as absence of evidence for superadditive synergism – not as proof of independent causal pathways. Nonetheless, all eight RERI point estimates were directionally consistent with sub-additive or null interaction, and confidence intervals excluded clinically meaningful synergism for the primary outcomes. What can be stated is that cycle-length risk information is present across metabolic strata, not concentrated in any one subgroup; this supports the practical utility of menstrual history inquiry regardless of metabolic screening results. The negative point estimates for long cycle × GDM RERI (range −0.21 to −0.39) may reflect partial pathway convergence: both long cycle and GDM can independently elevate PTB risk, partly through shared insulin-resistance pathways (40); women with GDM have already partially activated this shared pathway, attenuating the additional absolute risk increment attributable to cycle length in the GDM stratum. This interpretation is speculative; the operationally relevant finding is that cycle-length risk is present across all metabolic strata and is not amplified by co-existing GDM – supporting the utility of menstrual history inquiry regardless of metabolic screening status. The clinical implications of these findings are specific to the setting in which they were obtained and should be interpreted accordingly. In Chinese tertiary obstetric centers with the metabolic comorbidity burden observed here, GDM screening is universal and thyroid function testing is routine. Our data show that in this setting, a woman’s menstrual cycle length confers an additional, non-redundant 36–46% excess risk of PTB and LBW compared to women with normal cycle length, after accounting for metabolic comorbidities.

The population-attributable fractions (PTB 3.1%, LBW 3.9%) reflect the cycle-length-associated burden within this specific tertiary cohort and should not be extrapolated to community settings where GDM prevalence is substantially lower. Although the AUC increment was modest (ΔAUC 0.006–0.007; DeLong *p* > 0.10)—as expected for a single categorical marker added to an established model – the interpretation of this finding requires distinguishing two distinct clinical value propositions. AUC quantifies a model’s ability to rank individuals by risk probability, a metric optimized for scenarios where a clinician must discriminate between two individuals. Cycle length, by contrast, operates as a risk-enrichment signal: it identifies a population subgroup (long-cycle women, 10.2% of this cohort) that disproportionately contributes to PTB and LBW burden and that metabolic screening is structurally unable to detect, given that cycle length and GDM carry non-redundant risk information (RERI≈0; OR stability M1–M3). This distinction is analogous to the incremental value of family history in cardiovascular risk models: family history rarely improves AUC substantially, yet its systematic omission from clinical history-taking would leave a meaningful and addressable risk dimension undetected (41). The statistically significant IDI and continuous NRI confirm that the full model assigns systematically higher predicted probabilities to women who subsequently experienced these outcomes – establishing that the cycle-length signal carries genuine probabilistic information beyond the base metabolic model, even if its magnitude is insufficient to shift ROC discriminability (full results in **Supplementary Table 22**; **Supplementary Figure 12**). The actionable implication is not that cycle length enables precise individual-level risk prediction, but that it identifies a clinically accessible subgroup with independently elevated risk that would remain undetected by metabolic screening alone. This information could be ascertained antenatally through a single standardized question – before laboratory results are returned, without additional cost or infrastructure (42). These findings should be considered hypothesis-generating pending prospective validation; cycle length is a risk enrichment marker, not a diagnostic criterion, and should not independently drive intervention decisions.

This study has several important limitations. (i) Single-center tertiary referral cohort with GDM prevalence of 37.0%: this design introduces two distinct generalizability concerns that should be interpreted separately. First, referral bias may inflate the observed associations. Women with long menstrual cycles referred to a tertiary center are likely selected based on co-existing reproductive or metabolic complexity – precisely the factors that also increase PTB and LBW risk. This selection mechanism enriches the long-cycle group with higher-risk women compared to the unselected obstetric population, potentially upwardly biasing the observed aORs. The magnitude of this bias cannot be quantified from the present data; prospective community-based cohorts are needed to assess whether the aOR estimates of 1.36 (PTB) and 1.46 (LBW) are reproducible outside tertiary referral settings. Second, the high institutional GDM prevalence (37.0%) exceeds national estimates for China (approximately 14–17% by IADPSG criteria in community samples) by more than twofold. This affects generalizability at two levels. At the descriptive level, PAF estimates from this cohort (PTB 3.1%, LBW 3.9%) directly incorporate the local GDM prevalence and should not be extrapolated to community settings where GDM burden – and thus the baseline risk landscape – differs substantially. At the inferential level, the conditional OR for long cycle after adjustment for GDM may itself vary across GDM-prevalence settings. In a high-GDM environment, a larger proportion of the background PTB and LBW risk is already attributable to GDM-related pathways; adjusting for GDM therefore removes more shared variance, and the residual cycle-length OR may represent a narrower, more specific signal than would be observed in lower-GDM populations where metabolic pathways are less saturated. Whether the long-cycle OR in community settings would be larger (because less metabolic variance has been removed) or smaller (because referral bias is absent) cannot be determined from the present study. These two sources of uncertainty operate in opposite directions and partially offset each other, but neither can be dismissed. Replication in prospective community cohorts with lower GDM prevalence is necessary to characterize the transportability of these findings. Because the population-attributable fraction depends not only on the strength of the association between the exposure and the outcome but also on the prevalence of the exposure and of coexisting conditions, it is specific to the population in which it is estimated (43). In community obstetric settings, where the GDM burden is substantially lower than in this referral cohort, the fraction attributable to long cycles would be expected to differ and would need to be re-estimated directly rather than transported from these data. (ii) Menstrual phenotype data were collected by structured self-report at the intrapartum admission visit; the short interval from the last menstrual period substantially limits recall imprecision, and differential bias is implausible given that women were unaware of cycle length as a perinatal risk factor. Nonetheless, absence of objective validation (e.g., menstrual diary or app-based records) means measurement error cannot be fully excluded; non-differential misclassification would bias estimates toward the null, suggesting results are conservative. Prior validation work indicates that retrospectively recalled usual cycle length agrees moderately well with prospectively recorded cycles, with most error being random rather than systematic (44), further supporting a non-differential, toward-the-null interpretation of any residual misclassification in our setting. (iii) Pre-pregnancy BMI is the theoretically preferred confounder but was available for only 41.7% of women. Admission BMI was used in the primary analysis as a pragmatic proxy (98.4% complete), with acknowledged limitations: it may lie partly on the causal pathway between cycle length and PTB (potential mediator via gestational weight gain), or may introduce collider bias if unmeasured factors jointly affect gestational weight gain and PTB risk (see **Supplementary Figure 2**). Admission BMI showed high correlation with pre-pregnancy BMI (Pearson *r* = 0.796, Bland-Altman bias=5.15 kg/m^2^; **Supplementary Figure 13**), and MICE imputation of pre-pregnancy BMI (m=20 datasets) yielded long-cycle aOR 1.30 (95% CI 1.08–1.57) for PTB and aOR 1.40 (95% CI 1.14–1.72) for LBW (**Supplementary Table 4**), confirming directional consistency. Truncated IPW (95th percentile) yielded PTB aOR 1.47 and LBW aOR 1.54, consistent with the primary and MICE estimates (full IPW weight diagnostics in **Supplementary Table 25**; **Supplementary Figure 14**; note: the IPW analysis used n=7,380, the subset of the SA2 pre-pregnancy BMI complete cases (n=7,426) with valid stabilized weights after excluding 46 women with extreme propensity scores). All stable analytical approaches converge on long-cycle aOR 1.3–1.5 for both primary outcomes. A four-model comparison confirmed that this conclusion does not depend on how BMI was handled: the long-cycle association remained significant with no BMI adjustment, with admission BMI, with pre-pregnancy BMI in complete cases, and with imputed pre-pregnancy BMI (preterm birth aOR 1.28–1.43; low birth weight aOR 1.40–1.48; all *p* < 0.05; **Supplementary Table 5**), indicating the results are not an artefact of using admission rather than pre-pregnancy BMI. We therefore emphasize the treatment of BMI as a principal source of residual uncertainty. Because admission BMI is measured after the exposure, it may lie on the causal pathway to preterm birth (mediated through gestational weight gain) or act as a collider if unmeasured factors influence both gestational weight gain and preterm birth; under either interpretation, the fully adjusted estimates represent conditional associations rather than total causal effects. Although their stability across the alternative BMI specifications examined above argues against a measurement artifact, mediation or collider bias cannot be fully excluded with the available data. (iv) The mechanistic hormonal data most relevant to the proposed ovulatory-timing hypothesis – serial LH, FSH, and mid-luteal progesterone measurements – were not available in the EHR; all mechanistic inferences regarding luteal-phase insufficiency remain speculative. Several established perinatal risk factors were not available in the structured EHR — including prior preterm birth, maternal active and passive smoking, educational attainment, and non-ART fertility treatment — and so could not be included as covariates; residual confounding by these unmeasured factors cannot be excluded, and the conditional estimates should be read with this in mind. (v) Our four-source PCOS exclusion (ICD-10 coded diagnosis, clinician diagnosis, patient self-report, and structured clinical note review) identified and removed 245 women (1.3%). Residual concern is restricted to truly undiagnosed PCOS, principally Phenotype D (oligomenorrhea without overt hyperandrogenism). Because oligomenorrhea characterizes ~75–85% of women with PCOS, removal of diagnosed cases preferentially depleted the long-cycle group of its highest-PCOS-concentration stratum, substantially compressing residual enrichment. A formal quantitative bias analysis across 64 parameter combinations (PCOS prevalence 5–12.5%, undiagnosed fraction 50–80%, PCOS→outcome OR 1.5–3.0) confirmed robustness under a conservative 3× post-exclusion enrichment assumption: 63 of 64 PTB and 64 of 64 LBW scenarios retained a bias-corrected aOR >1.0. Under 2× enrichment, all 64 of 64 scenarios for both outcomes were retained (**Supplementary Table 24**; **Figure 6**). The monotonic restricted cubic spline dose–response without inflection at ≥45 days provides additional indirect evidence against confounding by a discrete undiagnosed PCOS subgroup. COVID-19 care-pathway changes during 2020–2022 were addressed by admission-year fixed effects; however, residual pandemic-related confounding cannot be excluded. The study was not prospectively registered. To preserve transparency, we explicitly distinguish between *a priori* elements (the three menstrual dimensions, the nested M1–M3 model sequence, BH-FDR correction, RCS, RERI, PAF, and five sensitivity analyses) and *post hoc* elements (birth-weight term-delivery analysis, incremental discrimination analyses, and all supplementary analyses labeled “EXPLORATORY”). Readers should weight the *a priori* findings accordingly. In particular, the short-cycle results are underpowered and should be considered exploratory and hypothesis-generating only; they do not support any clinical inferences regarding short cycles. Finally, caesarean delivery rates varied across cycle-length and metabolic subgroups (**Supplementary Table 26**): the short-cycle GDM group had a notably elevated caesarean rate (70.2% vs 51.0% in normal-cycle women without GDM/TF), which may reflect clinician recognition of androgen-excess phenotypes as an obstetric risk indicator. The potential for differential obstetric management to mediate part of the observed associations in the short-cycle GDM subgroup cannot be excluded and warrants investigation in prospective studies.

## Conclusions

In a large Chinese tertiary obstetric cohort, menstrual cycle length and metabolic comorbidities (GDM, thyroid dysfunction) provided independent risk information for adverse perinatal outcomes. A long cycle (>35 days) was independently associated with preterm birth (aOR 1.36) and low birth weight (aOR 1.46), with effect sizes unchanged after metabolic adjustment and without superadditive interaction with GDM or thyroid dysfunction. These findings do not redefine the biological architecture of perinatal risk but highlight an underutilized clinical signal that complements existing metabolic screening frameworks by capturing risk information not detected by routine GDM or thyroid testing. Our results are better characterized as demonstrating that menstrual cycle length retains prognostic value beyond routinely measured metabolic comorbidities, rather than establishing a distinct non-metabolic biological axis. Even in populations where GDM is systematically screened, menstrual cycle length provides additional risk information for preterm birth and low birth weight that metabolic screening does not capture. Because cycle length reflects the pre-pregnancy ovulatory interval, a single standardized question about usual cycle length, collected before conception or in early pregnancy, is a low-cost item that could be evaluated as a component of antenatal risk assessment; at delivery admission it would have limited predictive value, since gestational age is by then already determined. We frame this as a hypothesis-generating observation rather than a practice-changing recommendation. Prospective validation and hormonal mechanistic studies to characterize the biological basis of this association are the indicated next steps.

## Data Availability

The original contributions presented in the study are included in the article/**Supplementary Material**. Further inquiries can be directed to the corresponding authors.
