# Supplementary material for: Menstrual cycle length identifies perinatal risk information beyond routine metabolic antenatal screening: a retrospective cohort study of 17,792 deliveries
Source: Front Endocrinol (Lausanne). 2026 Aug 3;17:1888560. doi: 10.3389/fendo.2026.1888560 (PMC13478013; doi:10.3389/fendo.2026.1888560)
Supplement: Supplementary file 1 [file SupplementaryFile1.docx]

**Supplementary Figure S1.** Participant flow chart. Of 18,709 deliveries recorded in the electronic health records of Wuhan Children’s Hospital (January 2020–December 2024), 17,792 met all eligibility criteria after seven pre-specified sequential exclusions (total n = 917). Cycle length was available for 17,624 women (99.1%); the M3 complete-case analysis set comprised 17,336 women (97.4% of the eligible cohort) after exclusion of those missing cycle length or model covariates. The primary exposure contrast was long cycle (>35 days; n = 1,810) versus normal cycle (25–35 days; n = 15,613, reference); short cycle (<25 days; n = 201) was pre-specified as exploratory owing to limited power. Primary outcomes were preterm birth (n = 1,089; 6.1%) and low birth weight (n = 880; 4.9%). Sample sizes for sensitivity analyses SA1–SA5 are shown within the figure. Secondary analyses are reported separately: preterm-birth subtype classification (Table S2), size-for-gestational-age / fetal growth restriction (Table S14), and the four-model BMI comparison (Table S5).

**Figure S2.** Directed Acyclic Graph (DAG): causal position of BMI variables in the analysis. Nodes: Long cycle (exposure), PTB/LBW (outcomes), Pre-pregnancy BMI (theoretically correct confounder), Admission BMI (pragmatic proxy; circled to indicate ambiguous causal position), GDM, TF, Age, GWG (gestational weight gain), U (latent unmeasured factor). Key paths: Long cycle → PTB/LBW (primary path of interest); Long cycle → insulin resistance/HPO axis → GWG → Admission BMI (mediator path); GDM → GWG → Admission BMI; TF → Admission BMI; U → GWG and U → PTB/LBW (potential collider path activated by conditioning on Admission BMI). Pre-pregnancy BMI is a confounder (precedes both exposure and outcome). Admission BMI may act as a mediator (blocking part of the cycle-length effect on PTB) or as a collider (opening spurious paths through U), either of which biases M3 estimates in the conservative (downward) direction. The MICE analysis using imputed pre-pregnancy BMI addresses this by substituting the theoretically preferred confounder.

**Figure S3.** Missing data pattern. Bar chart of % missing for 12 key variables. Pre-pregnancy BMI had highest missingness (58.3%), motivating SA2. Primary outcomes had <0.1% missing. All menstrual phenotype data collected at the first antenatal booking visit (first trimester), before any outcome ascertainment.

**Figure S4.** GEE vs GLM sensitivity (SA1). Repeat pregnancies: 152/17,792 (0.9%); independence assumption confirmed. Near-identical GLM and GEE estimates confirm that clustering does not materially influence primary results. GEE serves as independence-assumption validity check, not a substantive robustness test.

**Figure S5.** Calibration plots for the base and full models. (A) Preterm birth; (B) low birth weight (n = 17,336). Women were grouped into deciles of predicted probability; points show observed event rate against mean predicted probability per decile for the base model (M3 covariates) and the full model (M3 + cycle category). The dashed diagonal denotes perfect calibration; curves are LOESS smoothers. Both models track the diagonal closely, indicating good calibration with no systematic mis-prediction after adding cycle category.

**Figure S6.** Decision-curve analysis for the base and full models. (A) Preterm birth; (B) low birth weight. Net benefit is plotted against threshold probability (0–30%) for the base model (M3 covariates) and the full model (M3 + cycle category). Adding cycle category produced a small positive increment in net benefit across low threshold probabilities (approximately 0–15%) with no loss elsewhere, consistent with a modest clinical-utility gain. Net benefit = (TP/N) − (threshold/(1−threshold)) × (FP/N).

**Figure S7.** Spearman correlation matrix. Cycle length shows near-zero Spearman correlations with PTB (ρ=0.00) and LBW (ρ=0.02); these low values reflect the binary nature of the outcomes and the categorical exposure, and are consistent with a modest but real association detectable in regression models. Period length shows near-zero correlations with PTB and LBW (ρ≈0.00–0.01)—graphical confirmation that the attributable burden is concentrated in the cycle-length dimension. Variables ordered by Ward.D2 hierarchical clustering.

**Figure S8.** RERI: long cycle (>35d) × GDM and × TF. No superadditive interaction for the primary outcomes: all long cycle × GDM RERI estimates were ≤0, and long cycle × TF estimates for PTB and LBW had 95% CIs crossing zero. Gestational-hypertension interaction with TF was positive, whereas the primary-outcome interactions were non-synergistic.

**Figure S9.** Period length supplementary analyses (three panels). (A) Forest plot (M1–M3): No period length category achieved FDR-significance for PTB or LBW. The nominally significant long period–gestational HTN association (aOR 1.58; p=0.024) did not survive FDR correction (p=0.171). (B) RCS dose–response: No significant omnibus association for PTB or LBW (P-overall >0.5 for both). Flat dose–response contrasts with monotonic cycle-length dose–response (Figure 3). (C) Stratified analysis: ORs for long period (>7d) within GDM and TF strata. Consistently null across all strata; P-int >0.2. Wide CIs reflect small long-period counts per stratum. All three panels are substantially underpowered (Table S1); interpret as hypothesis-generating only.

**Figure S10.** PAF comparison: long cycle (>35d) vs any abnormal cycle (long or short). Long cycle PAF: PTB 3.1% (CI 0.9–5.4%), LBW 3.9% (1.2–6.4%); any abnormal cycle PAF: PTB 3.7%, LBW 4.3%. Long period PAF (not shown separately): PTB 0.0% (−0.8% to 0.8%), LBW 0.0% (reported in text). The PAF differential between cycle-length categories and period-length should be interpreted with caution given period-length underpowering (Table S1). PAF by counterfactual standardisation, seed=42. Note: PAF estimates reflect a tertiary referral population; not extrapolable to community settings.

**Figure S11.** E-value visualisation. PTB: E-value 2.06 (CI lower bound 1.49); LBW: 2.28 (CI lower bound 1.64). An unmeasured confounder would need to be associated with both long cycle and the outcome by risk ratios exceeding these thresholds to fully explain the associations away. Computed from M3 fully adjusted ORs (VanderWeele & Ding 2017).

**Figure S12.** ROC curves for preterm birth (A) and low birth weight (B). Base model: GDM + TF + age + parity + gravidity + BMI + year. Full model: base + menstrual cycle category. ΔAUC = 0.0055 for PTB (DeLong p = 0.131) and ΔAUC = 0.0071 for LBW (DeLong p = 0.146). AUC increments of this magnitude are expected for a single categorical risk marker added to an established multivariate model; the primary value of cycle length lies in providing risk information not captured by metabolic screening (statistically significant IDI and NRI; Table S22) rather than standalone discriminative impact.

**Figure S13.** Bland–Altman plot: admission BMI vs pre-pregnancy BMI in n = 7,380 women with both measurements available. Bias = 5.15 kg/m² (systematic gestational weight gain); 95% limits of agreement 0.83–9.47 kg/m²; Pearson r = 0.796. Points coloured by cycle category confirm absence of differential measurement error across exposure groups (correlations consistent across Short, Normal, and Long cycle categories). This supports use of admission BMI as a valid proxy for pre-pregnancy BMI in the primary analysis, with multiple imputation (Table S4) as confirmatory sensitivity analysis.

**Figure S14.** Distribution of stabilised inverse probability weights (IPW) for pre-pregnancy BMI availability (SA2; n=7,380). Histogram of stabilised IPW weights with density overlay. Summary statistics: Mean=1.469, Median=0.587, IQR 0.512–0.681, Max=1,942.797, Kish effective sample size (ESS)=20 (0.3% of N=7,380). The extreme right tail (max weight 1,943) demonstrates severe weight instability in the untruncated IPW analysis. The dashed green line marks the 95th percentile (1.825); truncation at this threshold (Panel B of Table S25) eliminates the extreme weights and yields estimates (PTB aOR 1.47, LBW aOR 1.54) that converge with the primary analysis and MICE results. The Kish ESS of 20 indicates that the untruncated IPW analysis is effectively driven by only 20 observations, explaining the numerical instability of the untruncated PTB aOR (0.99). This figure motivates the use of truncated IPW (95th and 99th percentile) as the preferred IPW sensitivity analysis in place of untruncated stabilised weights.
