# Supplementary material for: Menstrual cycle length identifies perinatal risk information beyond routine metabolic antenatal screening: a retrospective cohort study of 17,792 deliveries
Source: Front Endocrinol (Lausanne). 2026 Aug 3;17:1888560. doi: 10.3389/fendo.2026.1888560 (PMC13478013; doi:10.3389/fendo.2026.1888560)
Supplement: Supplementary file 2 [file SupplementaryFile2.docx]

**Table S1. Post-hoc Power Analysis—Long Period Length vs. Preterm Birth**

| **Target OR** | **Baseline PTB (%)** | **N (long period >7d)** | **N (normal period 3-7d)** | **Power (%)** | **Interpretation** |
| --- | --- | --- | --- | --- | --- |
| 1.10 | 6.1 | 335 | 17,336 | 7.1 | Insufficient (<50%): negative result likely false negative |
| 1.20 | 6.1 | 335 | 17,336 | 15.3 | Insufficient (<50%): negative result likely false negative |
| 1.30 | 6.1 | 335 | 17,336 | 27.0 | Insufficient (<50%): negative result likely false negative |
| 1.36 | 6.1 | 335 | 17,336 | 35.1 | Insufficient (<50%): negative result likely false negative |
| POST-HOC POWER ANALYSIS: For the long-period analysis (n=335 exposed, baseline PTB rate 6.1%), statistical power to detect OR 1.20 = 15.3% and to detect OR 1.30 = 27.0%. The absence of a significant association between long period length and preterm birth should therefore be interpreted with caution, as the study was insufficiently powered to exclude modest effects of the magnitude observed for cycle length (OR 1.36). This non-finding does not constitute definitive evidence of ovulatory-axis specificity. | | | | | |

**Table S2. Preterm Birth Subtype Analysis: Spontaneous vs Indicated**

**Panel A. Distribution of PTB subtypes**

| **PTB subtype** | **N** | **%** |
| --- | --- | --- |
| Spontaneous PTB | 773 | 71.0 |
| Indicated PTB | 128 | 11.8 |
| Uncertain PTB | 188 | 17.3 |

**Panel B. PTB subtype by menstrual cycle category**

| **Cycle category** | **PTB subtype** | **N** | **%** |
| --- | --- | --- | --- |
| Normal (25–35 d) | Spontaneous PTB | 655 | 71.2 |
| Normal (25–35 d) | Indicated PTB | 110 | 12.0 |
| Normal (25–35 d) | Uncertain PTB | 155 | 16.8 |
| Short (<25 d) | Spontaneous PTB | 11 | 64.7 |
| Short (<25 d) | Indicated PTB | 1 | 5.9 |
| Short (<25 d) | Uncertain PTB | 5 | 29.4 |
| Long (>35 d) | Spontaneous PTB | 96 | 69.6 |
| Long (>35 d) | Indicated PTB | 16 | 11.6 |
| Long (>35 d) | Uncertain PTB | 26 | 18.8 |

**Panel C. Long cycle (>35 d) adjusted ORs by PTB subtype (M3 fully adjusted)**

| **PTB subtype** | **N (events)** | **aOR (95% CI)** | **P value** |
| --- | --- | --- | --- |
| Spontaneous PTB | 732 | **1.30 (1.04–1.62)** | **0.020** |
| Indicated PTB | 122 | 1.22 (0.68–2.05) | 0.468 |

Classification based on ICD-10-coded diagnoses in the EHR. Spontaneous PTB: preterm premature rupture of membranes, spontaneous labour onset, or spontaneous delivery in the absence of documented iatrogenic indicators. Indicated PTB: delivery without spontaneous labour, or delivery for documented maternal/fetal indications (severe pre-eclampsia, HELLP, placental abruption, fetal growth restriction). Uncertain: could not be distinguished from the EHR text. This classification was post-hoc and exploratory. Panel C models were adjusted for GDM, thyroid dysfunction, maternal age, parity, gravidity, admission BMI, and admission year; reference: normal cycle (25–35 d). Event counts in Panel C (732 spontaneous, 122 indicated) reflect complete-case covariate availability and are therefore lower than the totals in Panel A. The long-cycle association was statistically significant for spontaneous PTB (aOR 1.30, p = 0.020) but not for indicated PTB (aOR 1.22, p = 0.468); the indicated-PTB comparison was substantially underpowered.

**Table S3. Pre-pregnancy BMI Missingness—Logistic Regression Predictors**

| **Predictor** | **OR (95% CI) for pre-BMI missing** | **P value** |
| --- | --- | --- |
| Long menstrual cycle (>35d) | 1.04 (0.92-1.17) | 0.578 |
| GDM | 0.44 (0.41-0.48) | <0.001 |
| Thyroid dysfunction | 18.48 (16.63-20.57) | <0.001 |
| Maternal age (years) | 1.01 (1.00-1.02) | 0.061 |
| Parity: Para 1 | 1.81 (1.66-1.98) | <0.001 |
| Parity: Para >=2 | 1.90 (1.51-2.38) | <0.001 |
| adm_year | 0.51 (0.50-0.53) | <0.001 |
| Preterm birth (outcome) | 1.23 (1.03-1.47) | 0.026 |
| Low birth weight (outcome) | 1.20 (0.98-1.46) | 0.083 |
| Logistic regression; outcome = pre-pregnancy BMI missing (1) vs available (0). If key exposures (long_cycle) or outcomes (out_ptb, out_lbw) are significant predictors of missingness, the missing-completely-at-random (MCAR) assumption for SA2 is violated and SA2 results should be interpreted with additional caution. OR > 1 indicates the predictor is associated with higher probability of missing pre-BMI. | | |

**Table S4. BMI Sensitivity Analysis—Proxy Validity and Multiple Imputation**

**Panel A. Proxy Validity — Admission BMI vs Pre-Pregnancy BMI**

| **Subgroup** | **N** | **Pearson r** | **95% CI** | **Spearman rho** | **Mean diff (adm-pre)** | **SD of diff** |
| --- | --- | --- | --- | --- | --- | --- |
| Overall | 7,380 | 0.7958 | 0.7873–0.8040 | 0.7972 | 5.15 | 2.20 |
| Normal (25-35d) | 6,442 | 0.7902 |  | 0.7943 | 5.16 | 2.20 |
| Short (<25d) | 80 | 0.7759 |  | 0.7559 | 5.52 | 2.00 |
| Long (>35d) | 790 | 0.8243 |  | 0.8160 | 4.98 | 2.27 |
|  | 68 | 0.8639 |  | 0.8400 | 5.14 | 1.99 |
| Bland-Altman: Bias=5.15 kg/m² (admission higher than pre-pregnancy); 95% LoA=[0.83, 9.47]. Proportional bias check: r(diff,mean)=0.0617. Consistent Pearson r across cycle-length subgroups indicates absence of differential measurement error. | | | | | | |

**Panel B. Three-Way Comparison: aOR for Long Cycle on PTB and LBW**

| **Outcome** | **Analysis** | **N** | **aOR**  **(95% CI) Long cycle vs Normal** | **P value** | **Fraction of Missing Info†** |
| --- | --- | --- | --- | --- | --- |
| Preterm birth | Primary analysis (M3 fully adjusted, admission BMI, n=17,336) | 17,336 | 1.36 (1.12–1.64) | 0.002 | — |
| Low birth weight | Primary analysis (M3 fully adjusted, admission BMI, n=17,336) | 17,336 | 1.46 (1.18–1.78) | <0.001 | — |
| Preterm birth | SA2: complete-case pre-preg BMI (n=7,426) | 7,426 | 1.41 (1.06–1.86) | 0.016 | — |
| Low birth weight | SA2: complete-case pre-preg BMI (n=7,426) | 7,426 | 1.42 (1.02–1.93) | 0.030 | — |
| Preterm birth | MI (pre-pregnancy BMI, m=20) | 17,792 | 1.30 (1.08–1.57) | 0.006 | 0.003 |
| Low birth weight | MI (pre-pregnancy BMI, m=20) | 17,792 | 1.40 (1.14–1.72) | 0.001 | 0.002 |
| Preterm birth | IPW truncated (95th pct), SA2 (pre-preg BMI, n=7,380) | 7,380 | 1.47 (1.07–2.02) | 0.016 | — |
| Low birth weight | IPW truncated (95th pct), SA2 (pre-preg BMI, n=7,380) | 7,380 | 1.54 (1.08–2.20) | 0.018 | — |
| PANEL A — PROXY VALIDITY: Pearson correlation (r) and Bland-Altman statistics for admission BMI vs pre-pregnancy BMI in women with both measurements available. Differential bias across cycle-length categories would indicate non-random measurement error; consistent bias across strata supports validity of admission BMI as a proxy for metabolic status. †NOTE ON SAMPLE SIZES: The main SA2 complete-case analysis (Tables S6 and S4) uses n=7,426, defined as all women with pre-pregnancy BMI and complete M3 covariates. The IPW analysis in Panel A uses n=7,380 (46 fewer); these 46 women had pre-pregnancy BMI available but were additionally excluded from the IPW model due to missing stabilised weight computation (extreme weight truncation convergence). All results from both SA2 subsets (7,426 and 7,380) are directionally consistent and support the primary finding. PANEL B — THREE-WAY COMPARISON: aOR (adjusted odds ratio) for long cycle (>35d) vs normal cycle on preterm birth and low birth weight. Primary analysis uses admission BMI (n=17,336, 97.4% complete); SA2 uses pre-pregnancy BMI as a complete-case sensitivity analysis (n≈7,426, 41.7%); Multiple Imputation (MI) uses pre-pregnancy BMI imputed by MICE (m=20 imputed datasets, predictive mean matching, pooled by Rubin’s rule; imputation model included admission BMI, age, parity, gravidity, GDM, TF, cycle category, and both outcomes as auxiliary variables per Collins 2001); IPW truncated (95th pct) SA2 applies stabilised inverse probability weights truncated at the 95th weight percentile (1.83) for pre-pregnancy BMI availability to the SA2 complete cases. FMI = Fraction of Missing Information (MI only); indicates the proportion of variance attributable to imputation uncertainty (values >0.3 indicate high sensitivity to the imputation model). Consistency of aORs across all four approaches confirms that the primary finding is robust to BMI measurement strategy. | | | | | |

**Table S5. Robustness of the Long-Cycle Association to BMI Adjustment Strategy**

**Panel A. Preterm birth — long cycle (>35 d) vs normal (25–35 d)**

| **BMI handling** | **N** | **aOR (95% CI)** | **P** | **Events** |
| --- | --- | --- | --- | --- |
| 1. No BMI adjustment | 17,524 | 1.34 (1.11–1.62) | 0.002 | 1,061 |
| **2. + Admission BMI (primary analysis)** | **17,251** | **1.36 (1.13–1.65)** | **0.001** | **1,019** |
| 3. + Pre-pregnancy BMI (complete-case) | 7,314 | 1.43 (1.08–1.90) | 0.013 | 426 |
| 4. + Pre-pregnancy BMI (MICE, m=20) | 17,591 | 1.28 (1.06–1.54) | 0.010 | 1,072 |

**Panel B. Low birth weight — long cycle (>35 d) vs normal (25–35 d)**

| **BMI handling** | **N** | **aOR (95% CI)** | **P** | **Events** |
| --- | --- | --- | --- | --- |
| 1. No BMI adjustment | 17,524 | 1.44 (1.18–1.76) | <0.001 | 857 |
| **2. + Admission BMI (primary analysis)** | **17,251** | **1.48 (1.21–1.81)** | **<0.001** | **830** |
| 3. + Pre-pregnancy BMI (complete-case) | 7,314 | 1.43 (1.04–1.97) | 0.026 | 332 |
| 4. + Pre-pregnancy BMI (MICE, m=20) | 17,591 | 1.40 (1.14–1.71) | 0.001 | 868 |

*Each model adjusts for maternal age, parity, gravidity, admission year, GDM, and thyroid dysfunction, and differs only in how body-mass index is handled. Model 1 omits BMI entirely; Model 2 uses admission BMI (the primary analysis, ~98% complete); Model 3 uses pre-pregnancy BMI in complete cases only (pre-pregnancy BMI was missing for 58% of women, reducing the sample to ~7,300); Model 4 uses pre-pregnancy BMI imputed by multiple imputation by chained equations (predictive mean matching, m = 20, pooled by Rubin's rules). Short-cycle women were excluded, as in the main analysis. The long-cycle association with both preterm birth (aOR range 1.28–1.43) and low birth weight (aOR range 1.40–1.48) remained statistically significant across all four BMI-handling strategies, indicating that the findings are robust to the choice of BMI covariate and are not an artefact of using admission rather than pre-pregnancy BMI.*

**Table S6. Sensitivity Analyses SA2–SA5 — Long Cycle on Primary Outcomes**

| **Sensitivity analysis1** | **Outcome** | **N** | **OR (95% CI) for Long cycle** | **P value** |
| --- | --- | --- | --- | --- |
| SA2: Pre-preg BMI subset | Gestational hypertension | 7,426 | 1.06 (0.82-1.37) | 0.635 |
| SA2: Pre-preg BMI subset | Preterm birth | 7,426 | 1.41 (1.06-1.86) | 0.016 |
| SA2: Pre-preg BMI subset | Low birth weight | 7,426 | 1.42 (1.02-1.93) | 0.030 |
| SA2: Pre-preg BMI subset | Macrosomia | 7,426 | 1.33 (0.93-1.86) | 0.109 |
| SA3: Thyroid-free subset | Gestational hypertension | 11,753 | 0.92 (0.73-1.16) | 0.498 |
| SA3: Thyroid-free subset | Preterm birth | 11,753 | 1.27 (1.00-1.58) | 0.043 |
| SA3: Thyroid-free subset | Low birth weight | 11,753 | 1.40 (1.08-1.78) | 0.009 |
| SA3: Thyroid-free subset | Macrosomia | 11,753 | 1.38 (1.05-1.79) | 0.017 |
| SA4: Exclude ART (n=16909) | Gestational hypertension | 16,909 | 1.08 (0.89-1.30) | 0.428 |
| SA4: Exclude ART (n=16909) | Preterm birth | 16,909 | 1.32 (1.08-1.59) | 0.006 |
| SA4: Exclude ART (n=16909) | Low birth weight | 16,909 | 1.42 (1.15-1.75) | <0.001 |
| SA4: Exclude ART (n=16909) | Macrosomia | 16,909 | 1.23 (0.96-1.56) | 0.087 |
| SA5_HYPO: Clinical hypothyroidism only (n=16562) | Gestational hypertension | 16,562 | 1.09 (0.90-1.31) | 0.355 |
| SA5_HYPO: Clinical hypothyroidism only (n=16562) | Preterm birth | 16,562 | 1.35 (1.11-1.64) | 0.002 |
| SA5_HYPO: Clinical hypothyroidism only (n=16562) | Low birth weight | 16,562 | 1.40 (1.13-1.73) | 0.002 |
| SA5_HYPO: Clinical hypothyroidism only (n=16562) | Macrosomia | 16,562 | 1.25 (0.98-1.57) | 0.069 |
| SA2: pre-pregnancy BMI recorded (n=7,426 for cycle-length analyses; n=7,404 for period-length analyses in Table S17—the small difference reflects additional missing values in the period-length exposure within this subset). SA3: no thyroid dysfunction (n=11,753 for cycle-length analyses; n=11,724 for period-length analyses in Table S17—same reason). SA4: non-ART conceptions. SA5_HYPO: restricted to women without TF or with clinical hypothyroidism only (excludes hyperthyroidism [n=378], thyroiditis [n=311], other TF subtypes [n=258]); has_tf replaced by has_hypo (clinical hypothyroidism indicator). The reported SA5 analytic n (16,562) is lower than the simple subtraction of excluded TF subtypes from the full cohort (16,845) due to M3 complete-case restrictions (missing BMI and other covariates) within this subset. The primary analysis uses admission BMI rather than pre-pregnancy BMI because the latter was available in only 41.7% of women. Admission BMI reflects post-gestational-weight-gain status and is theoretically less ideal as a confounder; however, SA2 using pre-pregnancy BMI in the available subset shows consistent results, supporting that this choice does not materially affect the primary estimates. | | | | |

**Table S7. Combined 24-Test FDR Correction—Within-Dimension vs. All-Combined**

| **Exposure1** | **Outcome** | **OR_CI** | **P_raw** | **P-FDR** | **Sig.** | **P-FDR** | **Sig.** |
| --- | --- | --- | --- | --- | --- | --- | --- |
|  |  |  |  | **(within dimension)** | **(within)** | **(all combined)** | **(combined)** |
| Short (<25d) | Gestational hypertension | 0.78 (0.40-1.36) | 0.412 | 0.471 |  | 0.5 | ns |
| Long (>35d) | Gestational hypertension | 1.09 (0.90-1.30) | 0.364 | 0.471 |  | 0.5 | ns |
| Short (<25d) | Preterm birth | 1.49 (0.87-2.40) | 0.12 | 0.241 |  | 0.344 | ns |
| Long (>35d) | Preterm birth | 1.36 (1.12-1.64) | 0.001 | 0.006 | ** | 0.015 | * |
| Short (<25d) | Low birth weight | 1.32 (0.69-2.28) | 0.358 | 0.471 |  | 0.5 | ns |
| Long (>35d) | Low birth weight | 1.46 (1.18-1.78) | <0.001 | 0.002 | ** | 0.006 | ** |
| Short (<25d) | Macrosomia | 1.23 (0.57-2.30) | 0.56 | 0.56 |  | 0.623 | ns |
| Long (>35d) | Macrosomia | 1.25 (0.98-1.57) | 0.061 | 0.164 |  | 0.307 | ns |
| <11yrs | Gestational hypertension | 0.78 (0.58-1.05) | 0.11 | 0.439 |  | 0.344 | ns |
| 14 to15yrs | Gestational hypertension | 0.94 (0.80-1.10) | 0.425 | 0.51 |  | 0.5 | ns |
| >16yrs | Gestational hypertension | 0.69 (0.40-1.11) | 0.155 | 0.448 |  | 0.387 | ns |
| <11yrs | Preterm birth | 1.07 (0.76-1.45) | 0.698 | 0.698 |  | 0.698 | ns |
| 14 to 15yrs | Preterm birth | 0.86 (0.73-1.02) | 0.09 | 0.439 |  | 0.344 | ns |
| >16yrs | Preterm birth | 0.55 (0.30-0.92) | 0.036 | 0.432 |  | 0.24 | ns |
| <11yrs | Low birth weight | 0.76 (0.49-1.13) | 0.196 | 0.448 |  | 0.436 | ns |
| 14 to15yrs | Low birth weight | 0.89 (0.74-1.07) | 0.227 | 0.448 |  | 0.454 | ns |
| >16yrs | Low birth weight | 0.75 (0.42-1.24) | 0.299 | 0.448 |  | 0.498 | ns |
| <11yrs | Macrosomia | 1.22 (0.85-1.71) | 0.268 | 0.448 |  | 0.487 | ns |
| 14 to15yrs | Macrosomia | 1.09 (0.89-1.34) | 0.397 | 0.51 |  | 0.5 | ns |
| >16yrs | Macrosomia | 1.15 (0.63-1.92) | 0.627 | 0.684 |  | 0.66 | ns |
| Primary analysis treats each exposure dimension as an independent scientific hypothesis family for FDR correction. This table combines all M3 tests into a single family. Consistency across both methods confirms robustness to FDR family definition choice. BH = Benjamini–Hochberg FDR. Within-dimension: correction per exposure dimension (8 tests). Combined: correction across all 24 tests simultaneously. | | | | | | | |
|  |  |  |  |  |  |  |  |
|  |  |  |  |  |  |  |  |
|  |  |  |  |  |  |  |  |

**Table S8. Post-hoc Power Analysis—RERI Estimates: Long Cycle × GDM/TF**

| **Outcome** | **Interaction** | **RERI (95% CI)** | **SE** | **Direction** | **Power: RERI=0.3**  **(%)** | **Power: RERI=0.5**  **(%)** | **Power: RERI=0.7**  **(%)** |
| --- | --- | --- | --- | --- | --- | --- | --- |
| Gestational hypertension | Long cycle x GDM | -0.211 (-0.708, 0.285) | 0.253 | ≤ 0 (sub-additive/null) | 22.0 | 50.6 | 78.9 |
| Low birth weight | Long cycle x GDM | -0.246 (-0.821, 0.329) | 0.293 | ≤ 0 (sub-additive/null) | 17.6 | 39.9 | 66.5 |
| Macrosomia | Long cycle x GDM | -0.229 (-0.864, 0.407) | 0.324 | ≤ 0 (sub-additive/null) | 15.2 | 33.9 | 57.9 |
| Preterm birth | Long cycle x GDM | -0.385 (-0.957, 0.186) | 0.292 | ≤ 0 (sub-additive/null) | 17.7 | 40.3 | 67.0 |
| Gestational hypertension | Long cycle x TF | 0.586 (0.084, 1.087) | 0.256 | > 0 (super-additive) | 21.6 | 49.7 | 78.1 |
| Low birth weight | Long cycle x TF | 0.099 (-0.479, 0.677) | 0.295 | > 0 (super-additive) | 17.4 | 39.6 | 66.0 |
| Macrosomia | Long cycle x TF | -0.504 (-1.063, 0.055) | 0.285 | ≤ 0 (sub-additive/null) | 18.3 | 41.8 | 68.9 |
| Preterm birth | Long cycle x TF | 0.197 (-0.308, 0.703) | 0.258 | > 0 (super-additive) | 21.4 | 49.2 | 77.5 |
| POST-HOC POWER ANALYSIS: SE estimated from delta-method 95% CI: SE = (CI_upper - CI_lower) / (2 x 1.96). Power computed for two-sided alpha=0.05: Power = pnorm(\|RERI_target\|/SE - 1.96) + pnorm(-\|RERI_target\|/SE - 1.96). SIGN TEST: all 5/8 RERI point estimates are <= 0. Probability under H0 [P(negative)=0.5]: (0.5)^8 = 0.003906, one-sided binomial p = 0.3633. This directional consistency argument is INDEPENDENT of CI width and power limitations. CI PRECISION for primary outcomes: PTB x GDM upper CI = +0.19 (absolute excess risk < 1 percentage point above additive prediction); LBW x GDM upper CI = +0.33. POST-HOC POWER (PTB x GDM): 40.3% for RERI=0.5; 17.7% for RERI=0.3. Wider CIs for TF interactions and for macrosomia reflect smaller exposed-and-modifier-positive cell counts and correspond to lower post-hoc power; these estimates should be interpreted with appropriate caution. RERI: relative excess risk due to interaction (delta-method, interactionR package, VanderWeele & Knol 2014). GDM models adjusted for TF; TF models adjusted for GDM. | | | | | | | |

**Table S9. Baseline Characteristics Stratified by GDM Status**

| **Variable** | **Overall N=17,792** | **No GDM N=11,204** | **GDM N=6,588** | **P value** |
| --- | --- | --- | --- | --- |
| Maternal age, mean ± SD (years) | 31.3 ± 3.8 | 30.9 ± 3.7 | 32.0 ± 3.8 | <0.001 |
| **Parity, n (%)** |  |  |  | <0.001 |
| Primipara | 11,506 (64.7%) | 7,452 (66.5%) | 4,054 (61.5%) |  |
| Para 1 | 5,796 (32.6%) | 3,492 (31.2%) | 2,304 (35.0%) |  |
| Para ≥2 | 490 (2.8%) | 260 (2.3%) | 230 (3.5%) |  |
| Gravidity, mean ± SD | 2.0 ± 1.2 | 1.9 ± 1.1 | 2.1 ± 1.3 | <0.001 |
| Menarche age, mean ± SD (years) | 12.9 ± 1.0 | 13.0 ± 1.0 | 12.9 ± 1.0 | 0.007 |
| Cycle length, mean ± SD (days) | 31.0 ± 4.8 | 30.9 ± 4.7 | 31.2 ± 5.1 | <0.001 |
| **Cycle category, n (%)** |  |  |  | <0.001 |
| Normal (25–35d) | 15,613 (87.8%) | 9,899 (88.9%) | 5,714 (87.3%) |  |
| Short (<25d) | 201 (1.1%) | 133 (1.2%) | 68 (1.0%) |  |
| Long (>35d) | 1,810 (10.2%) | 1,060 (9.5%) | 750 (11.4%) |  |
| Period length, mean ± SD (days) | 5.7 ± 1.2 | 5.8 ± 1.2 | 5.6 ± 1.3 | <0.001 |
| Long period (>7d), n (%) | 339 (1.9%) |  |  |  |
| Admission BMI, mean ± SD (kg/m²) | 27.2 ± 3.4 | 27.0 ± 3.2 | 27.6 ± 3.7 | <0.001 |
| Pre-preg BMI (41.7%), mean ± SD | 22.2 ± 3.4 | 21.5 ± 3.0 | 22.8 ± 3.6 | <0.001 |
| Thyroid dysfunction, n (%) | 5,803 (32.6%) | 4,906 (43.8%) | 897 (13.6%) | <0.001 |
| ART conception, n (%) | 600 (3.4%) | 335 (3.0%) | 265 (4.0%) | <0.001 |
| Gestational hypertension, n (%) | 1,333 (7.5%) | 677 (6.0%) | 658 (10.0%) | <0.001 |
| Preterm birth, n (%) | 1,089 (6.1%) | 609 (5.4%) | 480 (7.3%) | <0.001 |
| Low birth weight, n (%) | 880 (4.9%) | 550 (4.9%) | 330 (5.0%) | 0.800 |
| Macrosomia, n (%) | 691 (3.9%) | 369 (3.3%) | 322 (4.9%) | <0.001 |

Mean ± SD or n (%). This is the original GDM-stratified baseline table, referenced from the main text in support of the cycle-length analysis. P values: Welch two-sample t-test (continuous) or Pearson chi-square test (categorical). Cycle category percentages are computed against the full cohort (N = 17,792); the three categories shown (Normal 15,613 + Short 201 + Long 1,810 = 17,624) exclude 168 women (0.9%) with missing cycle length. Notably, low birth weight prevalence did not differ by GDM status (No GDM 4.9% vs GDM 5.0%; p = 0.800), indicating that GDM-associated risk concentrates in macrosomia rather than low birth weight. GDM: gestational diabetes mellitus. ART: assisted reproductive technology.

**Table S10. GDM × Thyroid Dysfunction Cross-Tabulation**

**Panel A. GDM × thyroid-dysfunction cross-tabulation, n (%)**

| **GDM status** | **No thyroid dysfunction** | **Thyroid dysfunction** |
| --- | --- | --- |
| No GDM | 6,298 (35.4%) | 4,906 (27.6%) |
| GDM | 5,691 (32.0%) | 897 (5.0%) |

**Panel B. Outcome counts by metabolic subgroup (gestational-hypertension count verification)**

| **Metabolic subgroup** | **N (total)** | **Gest. HTN (n)** | **Gest. HTN (%)** | **PTB (n)** | **LBW (n)** |
| --- | --- | --- | --- | --- | --- |
| No GDM, No TF | 6,298 | 334 | 5.3 | 376 | 333 |
| TF only | 4,906 | 343 | 7.0 | 233 | 217 |
| GDM only | 5,691 | 563 | 9.9 | 410 | 272 |
| GDM + TF | 897 | 93 | 10.4 | 70 | 58 |

**Panel C. Annual prevalence of GDM, thyroid dysfunction, and gestational hypertension**

| **Year** | **N** | **GDM (%)** | **TF (%)** | **Gest. HTN (%)** |
| --- | --- | --- | --- | --- |
| 2020 | 3,063 | 37.6 | 19.9 | 5.1 |
| 2021 | 3,516 | 35.9 | 31.3 | 7.0 |
| 2022 | 3,295 | 33.4 | 31.8 | 7.9 |
| 2023 | 3,263 | 38.7 | 34.5 | 8.3 |
| 2024 | 4,650 | 38.9 | 41.2 | 8.6 |

Chi-square test for GDM × thyroid-dysfunction independence: χ² = 1717.1, p < 0.001. The lower-than-expected co-occurrence of GDM and thyroid dysfunction (GDM + thyroid dysfunction 897/17,792, 5.0%) reflects the distinct ascertainment pathways of the two conditions — thyroid dysfunction (predominantly clinical hypothyroidism, 83.7%) is frequently identified through pre-conception or first-trimester screening and dedicated endocrinology referral, whereas GDM is diagnosed at 24–28 weeks via universal OGTT — rather than a biological inverse relationship. Panel B reconciles the per-subgroup gestational-hypertension counts (334 + 343 + 563 + 93 = 1,333, matching Table 1). Panel C shows that GDM prevalence was stable across the study period while documented thyroid dysfunction rose (19.9% in 2020 to 41.2% in 2024), consistent with intensifying thyroid screening over time; admission-year fixed effects were included in all adjusted models.

**Table S11. Adjusted Risk Differences — Long Cycle vs Normal Cycle**

| **Outcome** | **N** | **ARD per 1,000 (95% CI)** | **P (bootstrap)** |
| --- | --- | --- | --- |
| Preterm birth | 17,501 | **18.30 (5.46 to 31.76)** | 0.012 |
| Low birth weight | 17,501 | **19.20 (5.68 to 30.57)** | <0.001 |

Adjusted risk differences (ARD) estimated by g-computation (marginal standardisation) from the fully adjusted M3 model (adjusted for GDM, thyroid dysfunction, maternal age, parity, gravidity, admission BMI, and admission year). Procedure: the M3 logistic model was fitted, outcome probabilities were predicted for the entire cohort under long cycle = 1 and long cycle = 0, and the ARD was computed as the difference in mean predicted probabilities, expressed per 1,000 women. 95% confidence intervals were obtained from 1,000 bootstrap resamples. Interpretation: among 1,000 women with a long cycle (>35 d) compared with a normal cycle (25–35 d), there are approximately 18 additional preterm births and 19 additional low-birth-weight infants. Reference: normal cycle (25–35 d).

**Table S12. RERI Decomposition—Component ORs and Cross-Verification: Long Cycle × GDM/TF**

| **Outcome** | **Modifier** | **N — Neither**  **(ref)** | **N — Long cycle only** | **N — Modifier only** | **N — Both** | **OR_E1 (long cycle only) 95% CI** | **OR_E2**  **(modifier only) 95% CI** | **OR_both**  **(both present) 95% CI** | **RERI_implicit†**  **(= OR_both − OR_E1 − OR_E2 + 1)** | **RERI_delta‡**  **(interactionR, 95% CI)** | **Cross-check \|Δ\|** | **Consistency** | **AP**  **(95% CI)** | **SI (95% CI)** |
| --- | --- | --- | --- | --- | --- | --- | --- | --- | --- | --- | --- | --- | --- | --- |
| Gestational hypertension | GDM | 594/9945 (6.0%) | 77/1042 (7.4%) | 562/5769 (9.7%) | 84/745 (11.3%) | 1.20 (0.93–1.54) | 1.52 (1.34–1.74) | 1.52 (1.16–1.96) | -0.211 | -0.211 (-0.708-0.285) | 0.000 | ✓ Consistent (<0.02) | -0.139 (-0.490-0.211) | 0.710 (0.308-1.638) |
| Preterm birth | GDM | 499/9945 (5.0%) | 78/1042 (7.5%) | 411/5769 (7.1%) | 57/745 (7.7%) | 1.55 (1.20–1.98) | 1.33 (1.15–1.53) | 1.49 (1.11–1.98) | -0.385 | -0.385 (-0.957-0.186) | 0.000 | ✓ Consistent (<0.02) | -0.258 (-0.689-0.173) | 0.562 (0.221-1.427) |
| Low birth weight | GDM | 459/9945 (4.6%) | 72/1042 (6.9%) | 277/5769 (4.8%) | 43/745 (5.8%) | 1.54 (1.18–1.99) | 1.01 (0.86–1.19) | 1.31 (0.93–1.80) | -0.246 | -0.246 (-0.821-0.329) | 0.000 | ✓ Consistent (<0.02) | -0.187 (-0.666-0.291) | 0.559 (0.127-2.463) |
| Macrosomia | GDM | 319/9945 (3.2%) | 46/1042 (4.4%) | 274/5769 (4.7%) | 45/745 (6.0%) | 1.37 (0.98–1.88) | 1.24 (1.04–1.48) | 1.39 (0.98–1.92) | -0.229 | -0.229 (-0.864-0.407) | 0.000 | ✓ Consistent (<0.02) | -0.165 (-0.656-0.327) | 0.629 (0.165-2.396) |
| Gestational hypertension | TF | 786/10551 (7.4%) | 97/1202 (8.1%) | 370/5163 (7.2%) | 64/585 (10.9%) | 0.93 (0.73–1.17) | 1.10 (0.96–1.27) | 1.62 (1.21–2.14) | 0.586 | 0.586 (0.084-1.087) | 0.000 | ✓ Consistent (<0.02) | 0.362 (0.130-0.594) | 19.198 (0.003-125911.867) |
| Preterm birth | TF | 660/10551 (6.3%) | 92/1202 (7.7%) | 250/5163 (4.8%) | 43/585 (7.4%) | 1.27 (1.00–1.58) | 0.85 (0.72–0.99) | 1.31 (0.93–1.79) | 0.197 | 0.197 (-0.308-0.703) | 0.000 | ✓ Consistent (<0.02) | 0.151 (-0.200-0.502) | 2.767 (0.119-64.308) |
| Low birth weight | TF | 506/10551 (4.8%) | 76/1202 (6.3%) | 230/5163 (4.5%) | 39/585 (6.7%) | 1.39 (1.08–1.78) | 0.90 (0.76–1.07) | 1.40 (0.98–1.94) | 0.099 | 0.099 (-0.479-0.677) | 0.000 | ✓ Consistent (<0.02) | 0.071 (-0.326-0.467) | 1.332 (0.249-7.109) |
| Macrosomia | TF | 431/10551 (4.1%) | 73/1202 (6.1%) | 162/5163 (3.1%) | 18/585 (3.1%) | 1.39 (1.06–1.80) | 0.94 (0.77–1.14) | 0.82 (0.48–1.30) | -0.504 | -0.504 (-1.063-0.055) | 0.000 | ✓ Consistent (<0.02) | -0.615 (-1.518-0.288) | NE |
| Component ORs (OR_E1, OR_E2, OR_both) were derived by the joint 4-level exposure method: a single factor variable encoding four mutually exclusive joint exposure states (Neither / Long cycle only / Modifier only / Both) was entered into logistic regression adjusted for age, parity, gravidity, admission BMI, admission year, and the other metabolic comorbidity as a covariate. This approach produces OR_E1, OR_E2, and OR_both with standard Wald 95% CIs that are independently interpretable. RERI VERIFICATION: RERI_implicit = OR_both - OR_E1 - OR_E2 + 1 (computed from joint ORs; no 95% CI reported as this is a cross-check quantity, not a primary estimate). RERI_delta = RERI from interactionR package using the delta method (VanderWeele & Knol 2014) applied to the interaction-term logistic model; this is the primary RERI estimate reported in Table 3. Cross-check column shows \|RERI_delta - RERI_implicit\|; values < 0.02 confirm arithmetic consistency between the two approaches. N columns show events/total (%). GDM models adjusted for TF; TF models adjusted for GDM. OR: odds ratio. CI: confidence interval. AP: attributable proportion. SI: synergy index. Long cycle: cycle length > 35 days. GDM: gestational diabetes mellitus. TF: thyroid dysfunction. | | | | | | | | | | | | | | |

**Table S13. Multiplicative Interaction (LRT) — Long Cycle × GDM and × TF**

| **Outcome** | **Exposure** | **Modifier** | **Chi2** | **df** | **P_LRT** |
| --- | --- | --- | --- | --- | --- |
| Gestational hypertension | long_cycle | has_gdm | 1.06 | 1 | 0.3035 |
| Gestational hypertension | long_cycle | has_tf | 5.62 | 1 | 0.0177 |
| Gestational hypertension | short_cycle | has_gdm | 0.24 | 1 | 0.6244 |
| Preterm birth | long_cycle | has_gdm | 2.75 | 1 | 0.0973 |
| Preterm birth | long_cycle | has_tf | 0.93 | 1 | 0.3360 |
| Preterm birth | short_cycle | has_gdm | 0.01 | 1 | 0.9047 |
| Low birth weight | long_cycle | has_gdm | 0.68 | 1 | 0.4090 |
| Low birth weight | long_cycle | has_tf | 0.22 | 1 | 0.6412 |
| Low birth weight | short_cycle | has_gdm | 0.28 | 1 | 0.5962 |
| Macrosomia | long_cycle | has_gdm | 0.76 | 1 | 0.3841 |
| Macrosomia | long_cycle | has_tf | 2.67 | 1 | 0.1020 |
| Macrosomia | short_cycle | has_gdm | 0.56 | 1 | 0.4531 |

**Table S14. Size-for-Gestational-Age Analysis (INTERGROWTH-21st): Long Cycle vs Normal Cycle**

**Panel A. Prevalence of SGA and FGR overall and by cycle category**

| **Group** | **N (z available)** | **SGA <10th, n (%)** | **FGR <3rd, n (%)** |
| --- | --- | --- | --- |
| Overall | 17,303 | 776 (4.48) | 168 (0.97) |
| Normal cycle (25–35 d) | 15,555 | 675 (4.34) | 145 (0.93) |
| Long cycle (>35 d) | 1,748 | **101 (5.78)** | 23 (1.32) |

**Panel B. Adjusted associations of long cycle with size-for-gestational-age outcomes (M3 fully adjusted)**

| **Outcome** | **Events** | **aOR / β (95% CI)** | **P value** |
| --- | --- | --- | --- |
| SGA (<10th centile) | 754 | **aOR 1.39 (1.11–1.73)** | **0.003** |
| FGR / severe SGA (<3rd centile) | 164 | aOR 1.40 (0.89–2.20) | 0.148 |
| Birthweight z-score (all) | — | **β −0.052 (−0.093 to −0.010)** | **0.015** |

**Panel C. Term deliveries only (≥37 weeks)**

| **Term deliveries only (≥37 wk)** | **Events** | **aOR / β (95% CI)** | **P value** |
| --- | --- | --- | --- |
| SGA (<10th centile) | 691 | **aOR 1.34 (1.07–1.69)** | **0.013** |
| Birthweight z-score | — | β −0.037 (−0.080 to +0.006) | 0.089 |

Birthweight-for-gestational-age z-scores were calculated using the INTERGROWTH-21st Newborn Birth Weight Standards (Villar et al., Lancet 2014). Gestational age was derived from the last menstrual period and date of delivery. SGA = z-score <10th centile (z < −1.2816); FGR / severe SGA = z-score <3rd centile (z < −1.8808). The INTERGROWTH-21st newborn birthweight standard spans 33+0 to 42+6 weeks; 219 infants <33 weeks fell outside the standard and were set to missing for this analysis. Of 17,792 women, 17,303 had an evaluable z-score (270 excluded for missing LMP, delivery date, sex, or birthweight, or implausible values). In this cohort the mean z-score was +0.18 (SD 0.88) and SGA prevalence (4.5%) was lower than the 10% expected in the standard population, while LGA (>90th) prevalence was 10.3%, consistent with prior reports that Chinese birthweights are higher than the INTERGROWTH-21st reference. Models were adjusted for GDM, thyroid dysfunction, maternal age, parity, gravidity, admission BMI, and admission year; reference: normal cycle (25–35 d); short-cycle women were excluded from these models, as in the main analysis. Long cycle was associated with higher odds of SGA overall and among term deliveries, and with a small reduction in birthweight z-score, indicating that the long-cycle birthweight signal reflects, in part, impaired fetal growth and not solely earlier delivery timing. The FGR (<3rd centile) comparison was underpowered (164 events).

**Table S15. Period Length Additive Interaction (RERI/AP/SI)—Long Period × GDM/TF**

| **Outcome** | **Interaction** | **OR_E1** | **OR_E2** | **OR_both** | **RERI** | **AP** | **SI** |
| --- | --- | --- | --- | --- | --- | --- | --- |
| Gestational hypertension | Long period x GDM | NE | NE | NE | -0.215 (-1.739-1.310) | -0.105 (-0.904-0.694) | 0.830 (0.208-3.306) |
| Preterm birth | Long period x GDM | NE | NE | NE | -0.901 (-1.879-0.077) | -1.238 (-3.474-0.998) | NE |
| Low birth weight | Long period x GDM | NE | NE | NE | -0.869 (-1.797-0.059) | -1.824 (-5.415-1.767) | NE |
| Macrosomia | Long period x GDM | NE | NE | NE | -0.537 (-1.346-0.272) | -2.042 (-8.478-4.394) | NE |
| Gestational hypertension | Long period x TF | NE | NE | NE | 1.622 (-0.013-3.257) | 0.569 (0.253-0.885) | 8.086 (0.452-144.674) |
| Preterm birth | Long period x TF | NE | NE | NE | 0.910 (-0.144-1.963) | 0.623 (0.223-1.023) | NE |
| Low birth weight | Long period x TF | NE | NE | NE | 0.346 (-0.701-1.393) | 0.303 (-0.434-1.039) | NE |
| Macrosomia | Long period x TF | NE | NE | NE | -0.197 (-0.906-0.513) | -0.881 (-5.358-3.596) | NE |

OR component columns (OR_E1/OR_E2/OR_both) are omitted: the joint 4-level logistic model was not estimable for the long period group (n=339 exposed). RERI, AP, and SI were estimated via delta method (interactionR). SI=NE where boundary convergence occurred; all 95% CIs include zero. Substantially underpowered (Table S1).

**Table S16. Period Length Population-Attributable Fractions (PAF)**

| **Outcome** | **Prevalence_pct** | **PAF_long_pct1** | **PAF_long_CI** | **PAF_abnormal_pct** | **PAF_abnormal_CI** |
| --- | --- | --- | --- | --- | --- |
| Gestational hypertension | 7.5 | 0.8 | 0.1%-1.5% | 0.8 | -0.0%-1.6% |
| Preterm birth | 6.1 | 0.0 | -0.8%-0.8% | 0.4 | -0.6%-1.4% |
| Low birth weight | 4.9 | 0.0 | -0.8%-1.1% | 0.2 | -0.9%-1.4% |
| Macrosomia | 3.9 | -0.9 | -1.5%--0.1% | -0.6 | -1.4%-0.3% |
| PAF = Population Attributable Fraction. long_period: >7d. period_abnormal: <3d or >7d. 95% CI from 999 bootstrap replicates. | | | | | |

**Table S17. Period Length Sensitivity Analyses (SA2 and SA3)**

| **Analysis1** | **Period category** | **Outcome** | **N** | **OR (95% CI)** | **P value** |
| --- | --- | --- | --- | --- | --- |
| SA2: Pre-preg BMI subset | Short (<3d) | Gestational hypertension | 7,404 | 0.67 (0.10-2.41) | 0.604 |
| SA2: Pre-preg BMI subset | Long (>7d) | Gestational hypertension | 7,404 | 1.05 (0.53-1.90) | 0.875 |
| SA2: Pre-preg BMI subset | Short (<3d) | Preterm birth | 7,404 | 2.60 (0.75-6.93) | 0.083 |
| SA2: Pre-preg BMI subset | Long (>7d) | Preterm birth | 7,404 | 0.57 (0.20-1.27) | 0.226 |
| SA2: Pre-preg BMI subset | Short (<3d) | Low birth weight | 7,404 | 2.42 (0.57-7.11) | 0.155 |
| SA2: Pre-preg BMI subset | Long (>7d) | Low birth weight | 7,404 | 0.46 (0.11-1.22) | 0.185 |
| SA2: Pre-preg BMI subset | Short (<3d) | Macrosomia | 7,404 | 1.68 (0.26-5.99) | 0.495 |
| SA2: Pre-preg BMI subset | Long (>7d) | Macrosomia | 7,404 | 0.21 (0.01-0.94) | 0.119 |
| SA3: Thyroid-free subset | Short (<3d) | Gestational hypertension | 11,724 | 1.32 (0.38-3.43) | 0.613 |
| SA3: Thyroid-free subset | Long (>7d) | Gestational hypertension | 11,724 | 1.10 (0.60-1.87) | 0.730 |
| SA3: Thyroid-free subset | Short (<3d) | Preterm birth | 11,724 | 2.50 (1.01-5.34) | 0.029 |
| SA3: Thyroid-free subset | Long (>7d) | Preterm birth | 11,724 | 0.69 (0.34-1.24) | 0.258 |
| SA3: Thyroid-free subset | Short (<3d) | Low birth weight | 11,724 | 1.89 (0.56-4.75) | 0.229 |
| SA3: Thyroid-free subset | Long (>7d) | Low birth weight | 11,724 | 0.87 (0.43-1.56) | 0.662 |
| SA3: Thyroid-free subset | Short (<3d) | Macrosomia | 11,724 | 1.00 (0.16-3.40) | 0.996 |
| SA3: Thyroid-free subset | Long (>7d) | Macrosomia | 11,724 | 0.54 (0.16-1.28) | 0.221 |
| Reference: Normal (3-7d). SA2: pre-pregnancy BMI. SA3: TF-free. | | | | | |

**Table S18. Secondary Outcomes—Amniotic Fluid Abnormality and Fetal Distress**

| **Exposure** | **Outcome** | **OR_CI** | **P_FDR** | **p_fdr_num** |
| --- | --- | --- | --- | --- |
| Short (<25d) | Amniotic fluid abnormality | 0.98 (0.67-1.41) | 0.934 | 0.933780211 |
| Long (>35d) | Amniotic fluid abnormality | 0.99 (0.87-1.12) | 0.934 | 0.933780211 |
| Short (<25d) | Fetal distress | 0.97 (0.61-1.46) | 0.934 | 0.933780211 |
| Long (>35d) | Fetal distress | 1.06 (0.91-1.22) | 0.934 | 0.933780211 |
| <11yrs | Amniotic fluid abnormality | 0.72 (0.57-0.90) | 0.013 | 0.012915880 |
| 14 to 15yrs | Amniotic fluid abnormality | 1.01 (0.91-1.11) | 0.865 | 0.865376786 |
| >16yrs | Amniotic fluid abnormality | 1.54 (1.19-1.96) | 0.005 | 0.004720493 |
| <11yrs | Fetal distress | 0.77 (0.60-0.99) | 0.092 | 0.091536668 |
| 14 to15yrs | Fetal distress | 0.97 (0.86-1.09) | 0.751 | 0.750701062 |
| >16yrs | Fetal distress | 1.29 (0.95-1.72) | 0.145 | 0.144633933 |
| Amniotic fluid abnormality and fetal distress were excluded from primary analysis: incidence rates were near-identical across cycle categories (<0.3% difference), and 75.2% of fetal distress cases co-occurred with amniotic fluid abnormality (Berkson bias). | | | | |

**Table S19. Population-Attributable Fractions (PAF)—Long Cycle and Any Abnormal Cycle**

| **Outcome** | **Prevalence_pct** | **PAF_long_pct1** | **PAF_long_CI** | **PAF_abnormal_pct** | **PAF_abnormal_CI** | **Attr_cases_long_exact** |
| --- | --- | --- | --- | --- | --- | --- |
| Gestational hypertension | 7.5 | 0.9 | -0.9% - 2.6% | 0.6 | -1.2% - 2.5% | 11.4 |
| Preterm birth | 6.1 | 3.1 | 0.9% - 5.4% | 3.7 | 1.4% - 6.0% | 32.5 |
| Low birth weight | 4.9 | 3.9 | 1.2% - 6.4% | 4.3 | 1.5% - 6.8% | 33.5 |
| Macrosomia | 3.9 | 2.4 | -0.1% - 5.3% | 2.6 | -0.1% - 5.6% | 16.1 |
| PAF = Population Attributable Fraction. Counterfactual standardization. 95% CI from 999 bootstrap. | | | | | | |

**Table S20. E-values for Unmeasured Confounding (M3 Fully Adjusted Estimates)**

| **Outcome1** | **Exposure** | **Prevalence** | **OR** | **CI** | **E_value** | **E_CI_lower** | **Interpretation** |
| --- | --- | --- | --- | --- | --- | --- | --- |
| Gestational hypertension | long_cycle | 7.5 | 1.09 | 0.91-1.31 | 1.41 | 1.00 | Weak |
| Preterm birth | long_cycle | 6.1 | 1.36 | 1.12-1.64 | 2.06 | 1.49 | Weak |
| Low birth weight | long_cycle | 4.9 | 1.46 | 1.18-1.78 | 2.28 | 1.64 | Moderate |
| Macrosomia | long_cycle | 3.9 | 1.25 | 0.98-1.57 | 1.79 | 1.00 | Weak |
| Gestational hypertension | cycle_abnormal | 7.5 | 1.06 | 0.89-1.26 | 1.31 | 1.00 | Weak |
| Preterm birth | cycle_abnormal | 6.1 | 1.37 | 1.14-1.63 | 2.07 | 1.53 | Moderate |
| Low birth weight | cycle_abnormal | 4.9 | 1.43 | 1.17-1.73 | 2.22 | 1.62 | Moderate |
| Macrosomia | cycle_abnormal | 3.9 | 1.24 | 0.99-1.55 | 1.79 | 1.00 | Weak |
| E-values computed from M3 fully adjusted OR estimates following VanderWeele & Ding (2017) Ann Intern Med. M3 covariates: age, parity, gravidity, admission BMI, year, GDM, TF. PCOS was excluded based on four operational criteria: (1) ICD-10-coded diagnosis in the EHR prior to or during the index pregnancy; (2) clinical diagnosis made by an obstetrician or endocrinologist during antenatal care at our institution; (3) patient-reported history of PCOS at the standardised admission interview; or (4) structured clinical note review identifying PCOS terminology or diagnostic criteria in the medical record. All women received systematic antenatal biochemical screening (thyroid function, glucose tolerance) and structured antenatal appointments at which reproductive history was reviewed; clinically evident PCOS first identified during pregnancy was captured through routine care pathways. Residual misclassification of subclinical undiagnosed PCOS — in women who never previously sought care — cannot be entirely excluded. However, the monotonic dose-response on the RCS curve (linear increase from 36 days upward, without inflection expected if a discrete PCOS subgroup were driving results) provides evidence against confounding by a binary subgroup. E-values (PTB: 2.06; LBW: 2.28) indicate an unmeasured confounder would need RR > 2.0 with both exposure and outcome to fully explain the associations. Menstrual history data (cycle length, period length, menarche age) were collected at the standardised intrapartum admission interview by trained midwives, prior to formal outcome recording. Because women were unaware of cycle length as a perinatal risk factor, differential recall bias affecting the primary associations is considered unlikely. However, the extended interval between the last menstrual period and the admission interview (typically 38–42 weeks) increases the potential for retrospective recall imprecision compared with early-pregnancy data collection. Non-differential misclassification of exposure would bias associations toward the null, suggesting reported estimates are conservative. Prospective validation using app-based menstrual tracking data remains a recommended future direction. | | | | | | | |

**Table S21. Sensitivity Analysis: Infection as a Potential Confounder**

**Panel A. Infection prevalence by cycle category**

| **Cycle category** | **N** | **Infection (n)** | **Infection (%)** |
| --- | --- | --- | --- |
| Normal (25–35 d) | 15,613 | 1,737 | 11.1 |
| Short (<25 d) | 201 | 22 | 10.9 |
| Long (>35 d) | 1,810 | 237 | 13.1 |

**Panel B. Long-cycle aOR stability after infection adjustment**

| **Outcome** | **Model** | **aOR (95% CI)** | **P value** |
| --- | --- | --- | --- |
| Preterm birth | M3 (without infection adjustment) | 1.36 (1.12–1.64) | 0.0015 |
| Preterm birth | **M3 + infection (sensitivity)** | **1.35 (1.12–1.63)** | 0.0018 |
| Low birth weight | M3 (without infection adjustment) | 1.46 (1.18–1.78) | <0.001 |
| Low birth weight | **M3 + infection (sensitivity)** | **1.45 (1.18–1.77)** | <0.001 |

Infection-related diagnoses were identified from ICD-10-coded EHR records (chorioamnionitis, urinary tract infection, genital-tract infection, and other maternal infections); overall prevalence 11.3% (n = 2,013). Infection was modestly more prevalent in the long-cycle group (13.1%) than the normal-cycle group (11.1%). If infection confounded the long-cycle–preterm-birth association, the long-cycle aOR would attenuate after adjustment; instead the estimates were essentially unchanged (PTB 1.36→1.35; LBW 1.46→1.45), indicating that the cycle-length associations are not materially confounded by documented infection. Non-differential misclassification of the infection variable would bias the infection coefficient toward the null but would not substantially bias the cycle-length coefficient unless infection were strongly associated with both long cycle and the outcome. Models adjusted for GDM, thyroid dysfunction, maternal age, parity, gravidity, admission BMI, and admission year.

**Table S22. Incremental Discriminative Analysis—AUC, IDI, and NRI**

| Outcome1 | AUC — Base model (95% CI)† | AUC — Full model (95% CI)‡ | ΔAUC | DeLong p | IDI (95% CI) | IDI p | NRI_continuous (95% CI) | NRI p |
| --- | --- | --- | --- | --- | --- | --- | --- | --- |
| Preterm birth | 0.5846 (0.5668–0.6024) | 0.5901 (0.5723–0.6078) | 0.0055 | 0.131 | 0.00069 (0.00022–0.00118) | 0.002 | 0.0700 (0.0269–0.1134) | <0.001 |
| Low birth weight | 0.5720 (0.5512–0.5929) | 0.5791 (0.5582–0.6000) | 0.0071 | 0.146 | 0.00088 (0.00038–0.00139) | <0.001 | 0.0785 (0.0315–0.1292) | 0.002 |
| Base model (†): logistic regression with age, parity, gravidity, admission BMI, admission year, GDM status, and thyroid dysfunction status. Full model (‡): base model + menstrual cycle category (Short/Normal/Long). AUC comparison by DeLong paired test (pROC package). IDI = Integrated Discrimination Improvement (Pencina 2008 Stat Med); measures the mean increase in predicted probability for events minus the mean increase for non-events when the new model is used. Bootstrap 95% CI (1,000 resamples, seed=42). NRI_continuous = continuous Net Reclassification Improvement (Pencina 2008); reflects proportional directional changes in predicted risk without category thresholds. Bootstrap 95% CI (1,000 resamples, seed=42). INTERPRETATION NOTE: AUC increments from individual binary risk factors are typically small (0.002-0.01) in obstetric risk models with multiple established predictors, even when the factor is independently associated (aOR ~1.3-1.5). The primary contribution of menstrual cycle length to perinatal risk assessment is its role as an independent risk marker — providing risk information not captured by GDM or thyroid dysfunction screening — rather than as a standalone predictor with large discriminative impact. The IDI and NRI results quantify the net directional reclassification and discrimination improvement achievable by adding cycle length to established metabolic predictors. | | | | | | | | |

**Table S23. Polycystic Ovary Syndrome (PCOS) Prevalence by Menstrual Cycle-Length Category, Before Four-Source Exclusion**

| **Menstrual cycle category** | **Women, n** | **PCOS, n** | **PCOS prevalence** |
| --- | --- | --- | --- |
| Short (<25 days) | 220 | 2 | 0.91% |
| Normal (25–35 days) | 16,233 | 81 | 0.50% |
| **Long (>35 days)** | **2,032** | **145** | **7.1%** |
| **Total (cycle recorded)** | **18,485** | **228** | **1.23%** |

PCOS was ascertained using the four-source approach described in Methods (ICD-10 coding, clinician diagnosis, patient self-report, and structured clinical-note review), applied to the full pre-exclusion cohort (N = 18,709). The tabulation is restricted to the 18,485 women with a recorded cycle category (224 with missing cycle length omitted). PCOS prevalence increased markedly across cycle-length categories, from 0.50% in normal-cycle women to 7.1% in long-cycle women (χ² = 653, df = 2, p < 0.001), a 14-fold enrichment. 64% of all PCOS cases occurred among long-cycle women, confirming that the four-source exclusion (n = 245) preferentially depleted the long-cycle stratum of its PCOS cases before analysis. This supports the quantitative bias analysis for residual undiagnosed PCOS (Table S24).

**Table S24. PCOS Quantitative Bias Analysis (QBA): Residual Undiagnosed PCOS Confounding**

**Panel A. Preterm Birth (Observed aOR = 1.36)**

| PCOS prevalence | Undiagnosed fraction | Residual undiagnosed (pop) | P(PCOS\|long cycle) | OR PCOS→outcome | Bias factor | Corrected aOR | Direction preserved |
| --- | --- | --- | --- | --- | --- | --- | --- |
| 5.0% | 50% | 2.5% | 7.5% | 1.5 | 1.028 | 1.32 | Yes |
| 5.0% | 50% | 2.5% | 7.5% | 2.0 | 1.055 | 1.29 | Yes |
| 5.0% | 50% | 2.5% | 7.5% | 2.5 | 1.081 | 1.26 | Yes |
| 5.0% | 50% | 2.5% | 7.5% | 3.0 | 1.107 | 1.23 | Yes |
| 5.0% | 60% | 3.0% | 9.0% | 1.5 | 1.033 | 1.32 | Yes |
| 5.0% | 60% | 3.0% | 9.0% | 2.0 | 1.065 | 1.28 | Yes |
| 5.0% | 60% | 3.0% | 9.0% | 2.5 | 1.097 | 1.24 | Yes |
| 5.0% | 60% | 3.0% | 9.0% | 3.0 | 1.128 | 1.21 | Yes |
| 5.0% | 70% | 3.5% | 10.5% | 1.5 | 1.039 | 1.31 | Yes |
| 5.0% | 70% | 3.5% | 10.5% | 2.0 | 1.076 | 1.26 | Yes |
| 5.0% | 70% | 3.5% | 10.5% | 2.5 | 1.113 | 1.22 | Yes |
| 5.0% | 70% | 3.5% | 10.5% | 3.0 | 1.148 | 1.18 | Yes |
| 5.0% | 80% | 4.0% | 12.0% | 1.5 | 1.044 | 1.30 | Yes |
| 5.0% | 80% | 4.0% | 12.0% | 2.0 | 1.087 | 1.25 | Yes |
| 5.0% | 80% | 4.0% | 12.0% | 2.5 | 1.128 | 1.21 | Yes |
| 5.0% | 80% | 4.0% | 12.0% | 3.0 | 1.168 | 1.16 | Yes |
| 7.5% | 50% | 3.8% | 11.2% | 1.5 | 1.041 | 1.31 | Yes |
| 7.5% | 50% | 3.8% | 11.2% | 2.0 | 1.081 | 1.26 | Yes |
| 7.5% | 50% | 3.8% | 11.2% | 2.5 | 1.120 | 1.21 | Yes |
| 7.5% | 50% | 3.8% | 11.2% | 3.0 | 1.158 | 1.17 | Yes |
| 7.5% | 60% | 4.5% | 13.5% | 1.5 | 1.049 | 1.30 | Yes |
| 7.5% | 60% | 4.5% | 13.5% | 2.0 | 1.097 | 1.24 | Yes |
| 7.5% | 60% | 4.5% | 13.5% | 2.5 | 1.143 | 1.19 | Yes |
| 7.5% | 60% | 4.5% | 13.5% | 3.0 | 1.188 | 1.14 | Yes |
| 7.5% | 70% | 5.2% | 15.8% | 1.5 | 1.057 | 1.29 | Yes |
| 7.5% | 70% | 5.2% | 15.8% | 2.0 | 1.113 | 1.22 | Yes |
| 7.5% | 70% | 5.2% | 15.8% | 2.5 | 1.166 | 1.17 | Yes |
| 7.5% | 70% | 5.2% | 15.8% | 3.0 | 1.217 | 1.12 | Yes |
| 7.5% | 80% | 6.0% | 18.0% | 1.5 | 1.065 | 1.28 | Yes |
| 7.5% | 80% | 6.0% | 18.0% | 2.0 | 1.128 | 1.21 | Yes |
| 7.5% | 80% | 6.0% | 18.0% | 2.5 | 1.188 | 1.14 | Yes |
| 7.5% | 80% | 6.0% | 18.0% | 3.0 | 1.245 | 1.09 | Yes |
| 10.0% | 50% | 5.0% | 15.0% | 1.5 | 1.055 | 1.29 | Yes |
| 10.0% | 50% | 5.0% | 15.0% | 2.0 | 1.107 | 1.23 | Yes |
| 10.0% | 50% | 5.0% | 15.0% | 2.5 | 1.158 | 1.17 | Yes |
| 10.0% | 50% | 5.0% | 15.0% | 3.0 | 1.207 | 1.13 | Yes |
| 10.0% | 60% | 6.0% | 18.0% | 1.5 | 1.065 | 1.28 | Yes |
| 10.0% | 60% | 6.0% | 18.0% | 2.0 | 1.128 | 1.21 | Yes |
| 10.0% | 60% | 6.0% | 18.0% | 2.5 | 1.188 | 1.14 | Yes |
| 10.0% | 60% | 6.0% | 18.0% | 3.0 | 1.245 | 1.09 | Yes |
| 10.0% | 70% | 7.0% | 21.0% | 1.5 | 1.076 | 1.26 | Yes |
| 10.0% | 70% | 7.0% | 21.0% | 2.0 | 1.148 | 1.18 | Yes |
| 10.0% | 70% | 7.0% | 21.0% | 2.5 | 1.217 | 1.12 | Yes |
| 10.0% | 70% | 7.0% | 21.0% | 3.0 | 1.282 | 1.06 | Yes |
| 10.0% | 80% | 8.0% | 24.0% | 1.5 | 1.087 | 1.25 | Yes |
| 10.0% | 80% | 8.0% | 24.0% | 2.0 | 1.168 | 1.16 | Yes |
| 10.0% | 80% | 8.0% | 24.0% | 2.5 | 1.245 | 1.09 | Yes |
| 10.0% | 80% | 8.0% | 24.0% | 3.0 | 1.318 | 1.03 | Yes |
| 12.5% | 50% | 6.2% | 18.8% | 1.5 | 1.068 | 1.27 | Yes |
| 12.5% | 50% | 6.2% | 18.8% | 2.0 | 1.133 | 1.20 | Yes |
| 12.5% | 50% | 6.2% | 18.8% | 2.5 | 1.195 | 1.14 | Yes |
| 12.5% | 50% | 6.2% | 18.8% | 3.0 | 1.255 | 1.08 | Yes |
| 12.5% | 60% | 7.5% | 22.5% | 1.5 | 1.081 | 1.26 | Yes |
| 12.5% | 60% | 7.5% | 22.5% | 2.0 | 1.158 | 1.17 | Yes |
| 12.5% | 60% | 7.5% | 22.5% | 2.5 | 1.231 | 1.10 | Yes |
| 12.5% | 60% | 7.5% | 22.5% | 3.0 | 1.300 | 1.05 | Yes |
| 12.5% | 70% | 8.8% | 26.2% | 1.5 | 1.094 | 1.24 | Yes |
| 12.5% | 70% | 8.8% | 26.2% | 2.0 | 1.183 | 1.15 | Yes |
| 12.5% | 70% | 8.8% | 26.2% | 2.5 | 1.266 | 1.07 | Yes |
| 12.5% | 70% | 8.8% | 26.2% | 3.0 | 1.344 | 1.01 | Yes |
| 12.5% | 80% | 10.0% | 30.0% | 1.5 | 1.107 | 1.23 | Yes |
| 12.5% | 80% | 10.0% | 30.0% | 2.0 | 1.207 | 1.13 | Yes |
| 12.5% | 80% | 10.0% | 30.0% | 2.5 | 1.300 | 1.05 | Yes |
| 12.5% | 80% | 10.0% | 30.0% | 3.0 | 1.387 | 0.98 | No |

**Panel B. Low Birth Weight (Observed aOR = 1.46)**

| PCOS prevalence | Undiagnosed fraction | Residual undiagnosed (pop) | P(PCOS\|long cycle) | OR PCOS→outcome | Bias factor | Corrected aOR | Direction preserved |
| --- | --- | --- | --- | --- | --- | --- | --- |
| 5.0% | 50% | 2.5% | 7.5% | 1.5 | 1.028 | 1.42 | Yes |
| 5.0% | 50% | 2.5% | 7.5% | 2.0 | 1.055 | 1.38 | Yes |
| 5.0% | 50% | 2.5% | 7.5% | 2.5 | 1.081 | 1.35 | Yes |
| 5.0% | 50% | 2.5% | 7.5% | 3.0 | 1.107 | 1.32 | Yes |
| 5.0% | 60% | 3.0% | 9.0% | 1.5 | 1.033 | 1.41 | Yes |
| 5.0% | 60% | 3.0% | 9.0% | 2.0 | 1.065 | 1.37 | Yes |
| 5.0% | 60% | 3.0% | 9.0% | 2.5 | 1.097 | 1.33 | Yes |
| 5.0% | 60% | 3.0% | 9.0% | 3.0 | 1.128 | 1.29 | Yes |
| 5.0% | 70% | 3.5% | 10.5% | 1.5 | 1.039 | 1.41 | Yes |
| 5.0% | 70% | 3.5% | 10.5% | 2.0 | 1.076 | 1.36 | Yes |
| 5.0% | 70% | 3.5% | 10.5% | 2.5 | 1.113 | 1.31 | Yes |
| 5.0% | 70% | 3.5% | 10.5% | 3.0 | 1.148 | 1.27 | Yes |
| 5.0% | 80% | 4.0% | 12.0% | 1.5 | 1.044 | 1.40 | Yes |
| 5.0% | 80% | 4.0% | 12.0% | 2.0 | 1.087 | 1.34 | Yes |
| 5.0% | 80% | 4.0% | 12.0% | 2.5 | 1.128 | 1.29 | Yes |
| 5.0% | 80% | 4.0% | 12.0% | 3.0 | 1.168 | 1.25 | Yes |
| 7.5% | 50% | 3.8% | 11.2% | 1.5 | 1.041 | 1.40 | Yes |
| 7.5% | 50% | 3.8% | 11.2% | 2.0 | 1.081 | 1.35 | Yes |
| 7.5% | 50% | 3.8% | 11.2% | 2.5 | 1.120 | 1.30 | Yes |
| 7.5% | 50% | 3.8% | 11.2% | 3.0 | 1.158 | 1.26 | Yes |
| 7.5% | 60% | 4.5% | 13.5% | 1.5 | 1.049 | 1.39 | Yes |
| 7.5% | 60% | 4.5% | 13.5% | 2.0 | 1.097 | 1.33 | Yes |
| 7.5% | 60% | 4.5% | 13.5% | 2.5 | 1.143 | 1.28 | Yes |
| 7.5% | 60% | 4.5% | 13.5% | 3.0 | 1.188 | 1.23 | Yes |
| 7.5% | 70% | 5.2% | 15.8% | 1.5 | 1.057 | 1.38 | Yes |
| 7.5% | 70% | 5.2% | 15.8% | 2.0 | 1.113 | 1.31 | Yes |
| 7.5% | 70% | 5.2% | 15.8% | 2.5 | 1.166 | 1.25 | Yes |
| 7.5% | 70% | 5.2% | 15.8% | 3.0 | 1.217 | 1.20 | Yes |
| 7.5% | 80% | 6.0% | 18.0% | 1.5 | 1.065 | 1.37 | Yes |
| 7.5% | 80% | 6.0% | 18.0% | 2.0 | 1.128 | 1.29 | Yes |
| 7.5% | 80% | 6.0% | 18.0% | 2.5 | 1.188 | 1.23 | Yes |
| 7.5% | 80% | 6.0% | 18.0% | 3.0 | 1.245 | 1.17 | Yes |
| 10.0% | 50% | 5.0% | 15.0% | 1.5 | 1.055 | 1.38 | Yes |
| 10.0% | 50% | 5.0% | 15.0% | 2.0 | 1.107 | 1.32 | Yes |
| 10.0% | 50% | 5.0% | 15.0% | 2.5 | 1.158 | 1.26 | Yes |
| 10.0% | 50% | 5.0% | 15.0% | 3.0 | 1.207 | 1.21 | Yes |
| 10.0% | 60% | 6.0% | 18.0% | 1.5 | 1.065 | 1.37 | Yes |
| 10.0% | 60% | 6.0% | 18.0% | 2.0 | 1.128 | 1.29 | Yes |
| 10.0% | 60% | 6.0% | 18.0% | 2.5 | 1.188 | 1.23 | Yes |
| 10.0% | 60% | 6.0% | 18.0% | 3.0 | 1.245 | 1.17 | Yes |
| 10.0% | 70% | 7.0% | 21.0% | 1.5 | 1.076 | 1.36 | Yes |
| 10.0% | 70% | 7.0% | 21.0% | 2.0 | 1.148 | 1.27 | Yes |
| 10.0% | 70% | 7.0% | 21.0% | 2.5 | 1.217 | 1.20 | Yes |
| 10.0% | 70% | 7.0% | 21.0% | 3.0 | 1.282 | 1.14 | Yes |
| 10.0% | 80% | 8.0% | 24.0% | 1.5 | 1.087 | 1.34 | Yes |
| 10.0% | 80% | 8.0% | 24.0% | 2.0 | 1.168 | 1.25 | Yes |
| 10.0% | 80% | 8.0% | 24.0% | 2.5 | 1.245 | 1.17 | Yes |
| 10.0% | 80% | 8.0% | 24.0% | 3.0 | 1.318 | 1.11 | Yes |
| 12.5% | 50% | 6.2% | 18.8% | 1.5 | 1.068 | 1.37 | Yes |
| 12.5% | 50% | 6.2% | 18.8% | 2.0 | 1.133 | 1.29 | Yes |
| 12.5% | 50% | 6.2% | 18.8% | 2.5 | 1.195 | 1.22 | Yes |
| 12.5% | 50% | 6.2% | 18.8% | 3.0 | 1.255 | 1.16 | Yes |
| 12.5% | 60% | 7.5% | 22.5% | 1.5 | 1.081 | 1.35 | Yes |
| 12.5% | 60% | 7.5% | 22.5% | 2.0 | 1.158 | 1.26 | Yes |
| 12.5% | 60% | 7.5% | 22.5% | 2.5 | 1.231 | 1.19 | Yes |
| 12.5% | 60% | 7.5% | 22.5% | 3.0 | 1.300 | 1.12 | Yes |
| 12.5% | 70% | 8.8% | 26.2% | 1.5 | 1.094 | 1.33 | Yes |
| 12.5% | 70% | 8.8% | 26.2% | 2.0 | 1.183 | 1.23 | Yes |
| 12.5% | 70% | 8.8% | 26.2% | 2.5 | 1.266 | 1.15 | Yes |
| 12.5% | 70% | 8.8% | 26.2% | 3.0 | 1.344 | 1.09 | Yes |
| 12.5% | 80% | 10.0% | 30.0% | 1.5 | 1.107 | 1.32 | Yes |
| 12.5% | 80% | 10.0% | 30.0% | 2.0 | 1.207 | 1.21 | Yes |
| 12.5% | 80% | 10.0% | 30.0% | 2.5 | 1.300 | 1.12 | Yes |
| 12.5% | 80% | 10.0% | 30.0% | 3.0 | 1.387 | 1.05 | Yes |

**Panel C. Enrichment Sensitivity Summary (2.0× / 3.0× / 5.0×)**

| **Outcome** | **Enrichment factor** | **Min corrected aOR** | **Median corrected aOR** | **Max corrected aOR** | **% scenarios aOR>1** |
| --- | --- | --- | --- | --- | --- |
| Low birth weight | 2 | 1.23 | 1.37 | 1.44 | 100.00 |
| Low birth weight | 3 | 1.05 | 1.29 | 1.42 | 100.00 |
| Low birth weight | 5 | 0.81 | 1.16 | 1.38 | 81.25 |
| Preterm birth | 2 | 1.14 | 1.28 | 1.34 | 100.00 |
| Preterm birth | 3 | 0.98 | 1.21 | 1.32 | 98.44 |
| Preterm birth | 5 | 0.75 | 1.08 | 1.29 | 67.19 |

This table systematically evaluates the impact of undiagnosed PCOS contamination on the observed long-cycle associations with PTB and LBW. Parameters: (1) Total PCOS prevalence in the source population (5–12.5%); (2) Fraction of PCOS cases remaining undiagnosed after four-source exclusion (50–80%); (3) OR for PCOS→outcome from published meta-analyses (Bahri Khomami et al. Nat Commun 2024). Bias correction formula: OR_corrected = OR_observed / Bias_Factor, where Bias_Factor = [P(PCOS|exposed) × (OR_PCOS−1) + 1] / [P(PCOS|unexposed) × (OR_PCOS−1) + 1] (Greenland 1996; Lash, Fox & Fink, Applying Quantitative Bias Analysis, 2009). PCOS enrichment in long-cycle group = 3.0× (primary analysis), with 2.0× and 5.0× as sensitivity bounds. 245 women with any PCOS documentation were excluded from the source population of 18037 using four sources (ICD-10, clinical diagnosis, self-report, text search). Direction preserved = corrected aOR > 1.0.

**Table S25. IPW Weight Diagnostics and Truncated IPW Analysis**

**Panel A. Stabilised IPW Weight Distribution**

| Statistic | Stabilised IPW |
| --- | --- |
| N (SA2 complete cases with valid IPW weights†) | 7380 |
| Mean | 1.469 |
| SD | 28.197 |
| Median | 0.587 |
| IQR (25th–75th) | 0.512–0.681 |
| Min | 0.461 |
| Max | 1942.797 |
| 95th percentile | 1.825 |
| 99th percentile | 3.730 |
| % weights > 5 | 0.57% |
| % weights > 10 | 0.41% |
| % weights > 20 | 0.28% |
| Effective sample size (Kish) | 20 (0.3% of N) |

**Panel B. Truncated IPW Comparison: aOR for Long Cycle on PTB and LBW**

| **Outcome** | **Analysis** | **N** | **aOR (95% CI)** | **P value** |
| --- | --- | --- | --- | --- |
| Preterm birth | SA2 unweighted (complete-case) | 7,380 | 1.39 (1.05–1.85) | 0.023 |
| Low birth weight | SA2 unweighted (complete-case) | 7,380 | 1.39 (1.01–1.92) | 0.045 |
| Preterm birth | IPW truncated at 95th (1.83) | 7,380 | 1.47 (1.07–2.02) | 0.016 |
| Low birth weight | IPW truncated at 95th (1.83) | 7,380 | 1.54 (1.08–2.20) | 0.018 |
| Preterm birth | IPW truncated at 99th (3.73) | 7,380 | 1.53 (1.07–2.18) | 0.018 |
| Low birth weight | IPW truncated at 99th (3.73) | 7,380 | 1.61 (1.07–2.42) | 0.022 |
| Preterm birth | IPW untruncated (stabilised) | 7,380 | 0.99 (0.43–2.26) | 0.984 |
| Low birth weight | IPW untruncated (stabilised) | 7,380 | 1.62 (1.05–2.49) | 0.028 |

Panel A: Distribution of stabilised inverse probability weights (IPW) for pre-pregnancy BMI availability. Extreme weights indicate strong predictors of missingness (thyroid dysfunction OR=18.5 for BMI missing, Table S3). Kish effective sample size (ESS) quantifies the loss of precision due to variable weighting; ESS << N indicates influential extreme weights. Panel B: Four IPW variants for long cycle aOR. Untruncated IPW yields numerical instability for PTB (untruncated PTB aOR=0.99, i.e. collapsed toward null) due to extreme weights driving the Kish ESS to 20; LBW untruncated aOR=1.62 due to extreme weights in the right tail. Truncation at the 99th and 95th percentiles produces estimates that converge towards the MICE estimate (PTB 1.30, LBW 1.40) and SA2 complete-case estimate (PTB 1.41, LBW 1.42). This convergence confirms that the divergence in untruncated IPW is driven by a small number of extreme weights rather than by genuine effect modification by missingness pattern. INTERPRETATION: All stable analytical approaches (primary admission BMI, SA2 complete-case, MICE, truncated IPW) converge on long-cycle aOR 1.3–1.5, supporting robustness of the primary finding to BMI measurement strategy. Sandwich (HC1) standard errors used for all weighted models.

**Table S26. Caesarean Delivery Rate by Menstrual Cycle Category and Metabolic Status**

| Cycle category | No GDM/TF | GDM Only | TF Only | GDM+TF |
| --- | --- | --- | --- | --- |
| Normal (25-35d) | 2851/5588 (51.0%) | 2681/4946 (54.2%) | 2060/4311 (47.8%) | 417/768 (54.3%) |
| Short (<25d) | 47/78 (60.3%) | 40/57 (70.2%) | 26/55 (47.3%) | 8/11 (72.7%) |
| Long (>35d) | 279/576 (48.4%) | 370/643 (57.5%) | 253/484 (52.3%) | 59/107 (55.1%) |
| Cesarean delivery rate by menstrual cycle category and metabolic status. Short cycle + GDM: 70.2% cesarean rate (40/57), markedly higher than normal cycle without GDM/TF (51.0%; 2851/5588). The elevated surgical delivery rate in the short-cycle GDM group may reflect clinician recognition of PCOS-like androgen excess as an obstetric risk indicator, representing a potential iatrogenic pathway contributing to adverse birth outcomes in this subgroup. | | | | |
